# Affinity purification of erythropoietin from cell culture supernatant combined with MALDI-TOF-MS analysis of erythropoietin *N*-glycosylation

**DOI:** 10.1038/s41598-017-05641-1

**Published:** 2017-07-13

**Authors:** David Falck, Markus Haberger, Rosina Plomp, Michaela Hook, Patrick Bulau, Manfred Wuhrer, Dietmar Reusch

**Affiliations:** 1 Center for Proteomics and Metabolomics, Leiden University Medical Center, Albinusdreef 2, 2333 ZA Leiden, The Netherlands; 2 Pharma Biotech Development Penzberg, Roche Diagnostics GmbH, 82377 Penzberg, Germany

## Abstract

Erythropoietin (EPO) is a heavily glycosylated hormone whose recombinant forms are used for treatment of anaemia. EPO glycosylation is important for its pharmacological properties. An analytical workflow, which can determine EPO glycosylation in an accurate and high-throughput fashion from cell culture supernatant (CCS) in approximately 24 h, offers the possibility to follow changes during production. To address this challenge, we present a complete workflow consisting of protein purification, glycan release, sialic acid derivatization, solid phase extraction, matrix-assisted laser desorption/ionization - mass spectrometry (MALDI-MS) analysis and MassyTools data processing. EPO purification from CCS by anti-EPO antibody coupled Sepharose beads yielded excellent purity with acceptable recovery and was free of glycoform bias. Glycosylation profiles obtained by MALDI-MS were highly comparable to those obtained with an established capillary gel electrophoresis–laser induced fluorescence method. Our method delivers accurate results for the analysis of changes of important glycosylation parameters, such as sialylation and number of N-acetyllactosamine units, for the time course of a fermentation. We could resolve differences in glycosylation between several CCS samples.

## Introduction

Erythropoietin (EPO) is a glycoprotein hormone best known for its role in the production of red blood cells, though it has multiple other physiological functions^1^. Its stimulation of erythropoiesis is therapeutically exploited, for example in the treatment of anaemia in chronic kidney disease and cancer^2, 3^. Consequently, recombinant human EPO is an important biopharmaceutical drug. Four forms, namely epoetin alfa, epoetin beta, epoetin delta and darbepoetin alfa, are distinguished mainly by their glycosylation (for example, in darbepoetin alfa, five amino acids are changed to generate two additional glycosylation sites)^4–6^. This and the fact that approximately half of EPO molecular weight is due to the glycan content, underlines the importance of glycan analysis for therapeutic EPO.

Typical glycan structures found on EPO’s three (epoetin) or five (darbepoetin) *N*-glycosylation sites are complex and mainly composed of tri- and tetra-antennary glycan structures with near complete galactosylation and high degrees of sialylation. Due to the faster clearance of galactose-terminated and low-antennary structures, sialic acid content and antennary structure represent critical quality attributes^4^. Additionally, distinction of *N*-acetylneuraminic acid (NeuAc) and *N*-glycolylneuraminic acid (NeuGc), the number of *N*-acetyllactosamine (LacNAc) repeats and the degree of *O*-acetylation (Ac) of sialic acids are relevant for pharmacokinetics^4^.

Although analytical release testing of biopharmaceuticals is performed on drug substances, which undergo multiple purification steps unrelated to the analysis, many discovery and development tasks require purification from cell culture supernatant (CCS). The possibility to analyze fermenter time course samples also opens new opportunities for biotechnological processes. For this purpose, elaborate glycoprotein purification schemes, such as multiple sequential chromatographic purifications, are too time consuming for fast analytical testing^7^. Moreover, considering the complexity of EPO, some extreme proteoforms may be lost by applying multiple sample purification steps. Affinity purification of EPO may represent a faster and more inclusive alternative. Several methods are available, varying in affinity partners and formats^8^. Mainly, anti-EPO antibodies are used as affinity partners, but others have been applied such as synthetic peptides, lectins and the erythropoietin receptor^8, 9^. Concerning formats, immobilization of the affinity partner to (monolithic) columns, well plates, magnetic and Sepharose beads have been reported^8^. Alternatively, recombinant EPO with a poly-histidine tag or expressed as a fusion protein, can be used for EPO purification from complex matrices^10, 11^. The large majority of characterization studies were done on EPO purified from human or animal body fluids whereas studies of recombinant EPO, purified from CCS, are rare^8, 12, 13^. To our best knowledge, no MS compatible purification strategy has demonstrated equivalence of the glycoform distribution before and after purification^14^.

Several methods for glycosylation analysis of purified EPO are available which have been recently reviewed^4, 15, 16^. Analysis of PNGaseF-released *N*-glycans is one of the most common approaches, though it does not address the site-specific microheterogeneity and completely ignores *O*-glycosylation. Among the approaches for released *N*-glycan detection, matrix-assisted laser desorption/ionization time-of-flight mass spectrometry (MALDI-TOF-MS) is frequently used, due to its high throughput (HT) and high information content. Though some reports have introduced reducing end derivatization in order to allow additional analysis by liquid chromatography with fluorescence detection (LC-FLD)^17, 18^, MALDI-MS analysis of non-derivatized glycans omits the intrinsic bias and reduced accuracy associated with this additional sample preparation step^19, 20^. However, derivatization of sialic acids can be useful to neutralize the charge and reduce the known instability of sialic acid containing glycans, observed in the reflectron mode of time-of-flight mass detection^21^. This phenomenon is often circumvented by desialylation prior to MALDI-MS and parallel analysis of sialylation by complementary techniques^17^. Another alternative is permethylation, but this derivatization technique suffers from the presence of satellite peaks, mainly due to incomplete or unwanted additional methylation^22^. All MALDI-MS methods for EPO glycosylation analysis described in literature report quite extensive sample preparation protocols, often featuring separate steps for desalting, protein removal, removal of excess label and even fractionation either by anion exchange or normal phase (liquid chromatography) LC^7, 17–19^.

**Figure 1:**
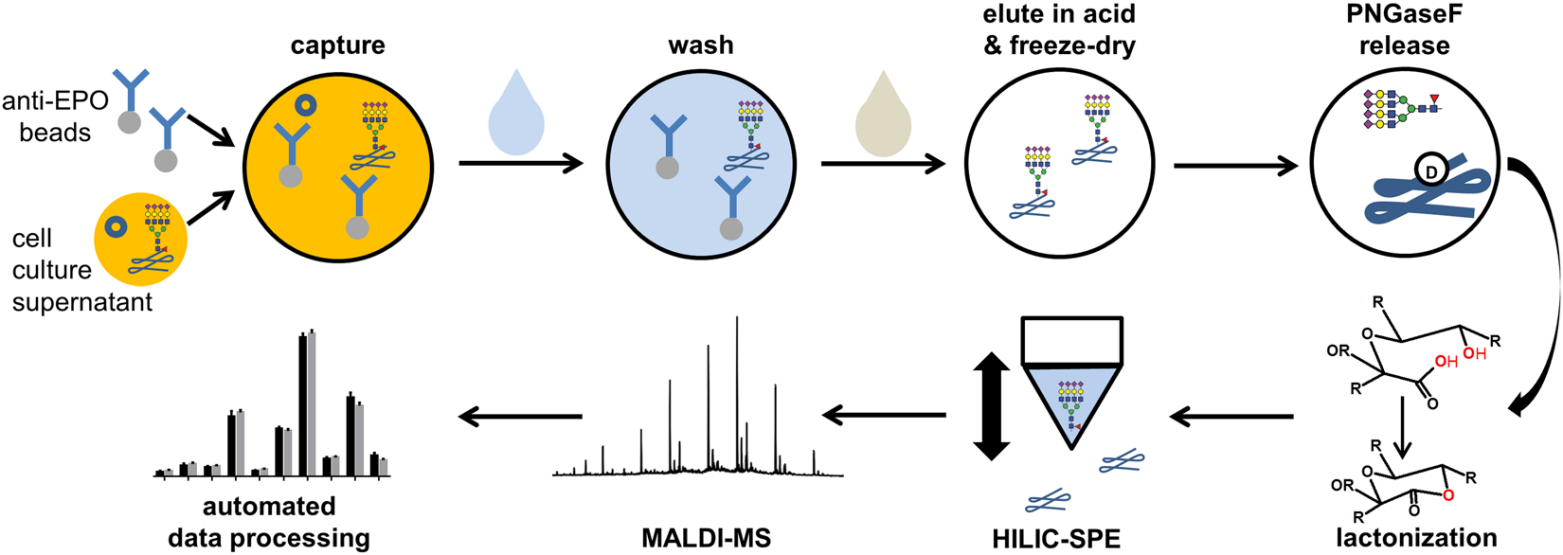
The CCS was mixed and incubated with anti-EPO beads in a 96-well filter plate. After washing, pure proteins were eluted in HCl and freeze dried. Glycans were prepared by PNGaseF release without additional denaturation, lactonization for stabilization and HILIC-SPE. MALDI-TOF-MS analysis in reflectron positive mode yielded high quality spectra which were automatically quantified with MassyTools.

Here, we report an HT workflow for EPO glycosylation analysis consisting of (1) EPO affinity purification, based on Sepharose bead-linked anti-EPO antibodies, from CCS, (2) efficiently stabilizing and easily interpretable sialic acid derivatization, (3) straightforward and easily automatable sample preparation, (4) high sensitivity and high resolution MALDI-TOF-MS analysis and (5) automated data processing (see Fig. 1). This approach could be used for analysis of recombinant EPO glycosylation. We envision that the method can provide fast feedback during discovery and development, especially where regular purification schemes are complex or not yet developed. Additionally, our method presents an alternative analysis pathway which may be able to pinpoint whether changes observed in the drug substance arise from changes in production or deviations in purification.

## Materials and Methods

### Samples and chemicals

Sepharose beads coupled to anti-erythropoetin antibodies (beads), erythropoetin reference standard (EPO standard) and CCS containing erythropoetin were kindly provided by Hoffmann-La Roche (Roche Diagnostics, Penzberg, Germany). Polyclonal antibodies from sheep raw serum were purified by delipidation, ammonium sulfate precipitation and ion exchange chromatography. The resulting antibodies were partially purified by immunoaffinity chromatography using EPO-sepharose. 10 mg of purified anti-erythropetin-antibodies were than coupled to 1 ml of NHS-activated Sepharose 4 Fast flow Beads following the instructions manual 71-5000-14 AD (GE Healthcare Life Sciences, Chicago, USA). The erythropoietin producing Chinese hamster ovary cell line was cultivated in chemically defined, animal component-free growth media. Samples were taken daily. The CCS sample was stored at −20 °C for later analysis. The 10 CCS samples, that were compared, were sampled at different time points from the same fermenter run. Beads were stored as a slurry containing 60% (v/v) of a mixture of phosphate-buffered saline (PBS) with 0.1% sodium azide (bead buffer). Furthermore, Peptide-*N*-glycosidase F (PNGaseF) was bought at Roche Diagnostics (Mannheim, Germany). 1-ethyl-3-(3-(dimethylaminopropyl)carbodiimide hydrochloride (EDC) was purchased from Fluorochem (Hadfield, United Kingdom). The following solvents and acids were from Merck KGaA (Darmstadt, Germany) and of analytical grade: ethanol, trifluoroacetic acid (TFA) and hydrochloric acid (HCl). Acetonitrile (ACN) was ordered at Biosolve (Valkenswaard, The Netherlands). Deionized water was generated with a Purelab ultra (ELGA Labwater, Ede, The Netherlands). All other chemicals were acquired in at least analytical quality from Sigma-Aldrich (Steinheim, Germany).

### Affinity purification

40 µL beads (100 µL slurry) were mixed with the sample in a 96-well filter plate (0.65 µm hydrophilic low protein binding Durapore membrane, Merck KGaA, Darmstadt, Germany). 5 µg EPO were applied unless otherwise stated, amounting to 180 µL of CCS or 2.5 µL of the EPO standard. Incubation at room temperature for an hour on a shaker ensured interaction between EPO and the antibodies. After capturing, the supernatant was eluted and the beads were washed three times with PBS and three times with water by centrifugation at 50 g. Elution was achieved by applying twice 100 µL 100 mM HCl which was eluted each at 500 g after 5 min vigorous shaking. The eluates were evaporated to dryness by freeze-drying in a centrifugal vacuum concentrator set to 60 °C (RVC2–25COplus, Martin Christ Gefriertrocknungsanlagen, Osterode am Harz, Germany).

To assess the purity and estimate recovery, the purification was done in triplicate and compared by sodium dodecylsulfate - polyacrylamide gel electrophoresis (SDS-PAGE) to a negative control (blank; no EPO added to the purification) and 1 µg, 2 µg and 5 µg of the EPO standard. For molecular weight determination a Novex Sharp Pre-Stained Protein Standard (Life Technologies, Paisley, UK) was taken along. SDS-PAGE was performed on a NuPage 4–12% Bis-Tris SDS-PAGE gel in MES SDS running buffer (both Life Technologies) after reduction for 10 min at 100 °C in Laemmli buffer with 1.3% β-mercaptoethanol^23^. Staining was achieved with Coommassie G-250 SimplyBlue SafeStain (Life Technologies).

Recovery was mainly assessed by a bicinchoninic acid (BCA) assay^24^. A micro BCA™ protein assay kit (ThermoFischer Scientific, Rockford, IL, USA) was used according to supplier specifications with one exception: the EPO standard was used for the calibration curve instead of bovine serum albumin.

### PNGaseF digestion

Freeze-dried eluates were reconstituted in 10 µL of a PBS solution containing 1% NP-40 and 0.5 U PNGaseF. After careful shaking for 10 min at room temperature, glycan release was achieved by incubating for 18 h at 37 °C.

In cases where the affinity purification was not performed, e.g. where the EPO standard was directly analysed, denaturation was achieved by incubation in 100 mM HCl or FA for 15 min at room temperature and subsequent freeze-drying (as described above). At least 75% (v/v) of the acid solution should be added to the sample and in strongly buffered samples the pH should be checked to be around or below 3. For immunoglobulin G glycosylation analysis, it has already been shown that acid denaturation is equally efficient as urea denaturation, reduction and alkylation^23^. Therefore, we investigated whether the dedicated detergent-assisted heat denaturing step, often employed in literature, was necessary for our analysis. We used a protocol in which the dried eluates from affinity purification were reconstituted in 5 µL 2% SDS for 5 min by vigorous shaking^21^. Afterwards, samples were incubated for 10 min at 60 °C. After cooling to room temperature, an equal volume of the NP-40/PGNaseF mixture was added and glycan release protocol continued as described above (notwithstanding protein:enzyme ratio changes).

### Derivatization, glycan enrichment and mass spectrometric analysis

Sialic acids were derivatized by ethyl esterification similarly to a previously described protocol^21^. In short, 100 µL of ethanol containing 250 mM each of EDC and 1-hydroxybenzotriazole hydrate were added for protein precipitation and derivatization. After incubation at 37 °C for one hour, 110 µL of ACN were added. From this solution, released and derivatized glycans were captured with in-house prepared hydrophilic interaction liquid chromatography – solid phase extraction (HILIC-SPE) pipet tips^21^. In brief, the pipet tips filled with a cotton thread were washed three times with 20 µL water and equilibrated three times with 20 µL of 85% aqueous ACN. After capture by 20 times filling and emptying, the pipet tips were washed three times each with 20 µL of 85% aqueous ACN with and without 1% TFA. Finally, glycans were eluted in 10 µL water.

The MALDI-TOF-MS experiments were conducted with the same instrumentation and settings as reported earlier^20^. Samples were prepared by mixing 4 µL of sample with 1 µL of matrix on a MALDI target plate 800/384 MTP AnchorChip (Bruker Daltonics, Bremen, Germany). The matrix consisted of a 9:1 mixture of 2,5-dihydroxybenzoic acid and 2-hydroxy-5-methoxybenzoic acid, sold as superDHB, and was dissolved at 5 mg/mL in 50% aqueous ACN containing 1 mM sodium hydroxide. Spectra were acquired on an ultrafleXtreme (Bruker Daltonics) between *m/z* 1000 and *m/z* 5000 in reflectron positive mode. Acceleration voltage was set to 25 kV and extraction delay to 140 ns. Spectra were accumulated with 20000 laser shots at 1000 Hz using a random walk algorithm and 200 shots per raster spot.

### Data analysis

The highest quality spectrum of the EPO standard and the EPO purified from CCS each was manually inspected for signals. These were assigned tentative compositions and glycoforms based on accurate mass measurements and known EPO glycosylation^4^. From these tentative compositions a calibration list and an analyte list were generated to be used in automated data (pre-)processing by MassyTools (version 1.8.1.2)^25^. With the help of this program, the spectra were internally calibrated and background corrected relative abundances of the analytes were extracted. Based on analyte quality criteria, analytes were selected for inclusion in the final relative quantitation. Analytes (1) had to be above a signal-to-noise (S/N) of 9 in at least 11 of 12 measurements, (2) have an average mass error of between −10 ppm and 10 ppm, (3) show an average isotopic pattern quality (IPQ) score ≤0.10 and (4) contribute ≥0.20% to the initial profile (equivalent to ca. ≥0.25% after analyte curation) to be considered for quantitation.

### Capillary gel electrophoresis – laser induced fluorescence

Unlabelled released glycans of the EPO standard were prepared in the same fashion as described above, except that they were additionally desialylated by sialidase treatment in parallel to the glycan release. Desialylated glycans were detected by capillary gel electrophoresis – laser induced fluorescence (CGE–LIF) as described earlier^26^. In brief, dried glycans were labelled with 8-aminopyrene-1,3,6-trisulfonic acid (ATPS) and purified by SPE. After addition of a size standard, samples were run on a DNA analyser (3730 DNA Analyzer, Applied Biosystems, Thermo Fisher Scientific).

## Results and Discussion

**Figure 2:**
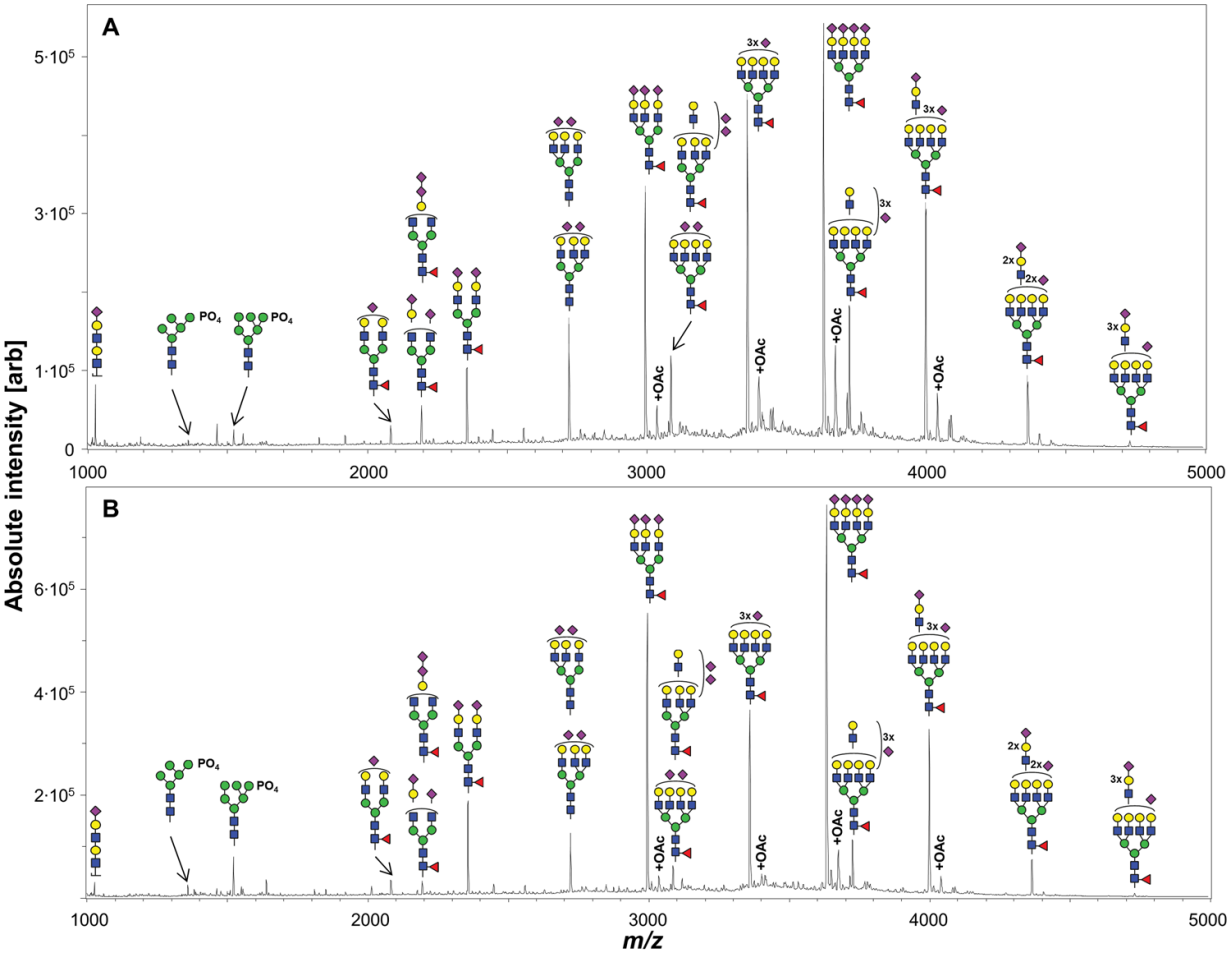
Representative MALDI-TOF-MS spectra of EPO *N*-glycans. (**A**) A spectrum of the EPO standard. It should be noted that the antenna isomerism indicated for the partially sialylated tri-antennary glycan also applies to all other tri-antennary structures. In addition, there may be minor contributions of bi-antennary structures with one LacNAc repeat. The tetra-antennary glycans have significant contributions of tri-antennary structures with one additional LacNAc repeat, as exemplified for the glycan with two NANA and 4 LacNAc units. (**B**) The major *N*-glycans detected for EPO purified from CCS were very similar to those of the EPO standard albeit in different relative abundances. 2× and 3× gives the incidence of the monosaccharide or antenna to the right or below. The brackets signify that the exact positions of substituents are unclear. The *N*-acetylneuraminic acids to the right of the vertical brackets may cap an antenna or LacNAc-repeat.

In this study, we developed a workflow for the accurate relative quantitation of EPO *N*-glycans directly isolated from cell culture supernatant. The analysis of released *N*-glycans by MALDI-TOF-MS is based on a recently introduced protocol which requires relatively few hands-on steps and is additionally very suitable for multiplexing and automation^21, 27^. MALDI-TOF-MS spectra of an EPO standard and EPO purified from CCS (see Fig. 1 and Material and Methods) are displayed in Fig. 2.

### Structure assignment

The 9 most abundant compositions in both the EPO standard and the purified EPO from CCS contained 2 to 6 LacNAc units, between 2 and 4 α2,3-linked sialic acids and were singly fucosylated. Individual glycoforms of these compositions arose from the decoration with between 0 and 3 sialic acid *O*-acetylations. In the following, we use compositions where *O*-acetylation variants are summed and glycoforms where individual *O*-acetylation variants are considered. The majority of sialic acids were *N*-acetylneuraminic acids (NANA), but many major compositions also showed a form where one NANA was replaced with an *N*-glycolylneuraminic acid (NGNA). Minor species included phosphorylated high mannose type glycans and compositions with equal amounts of hexoses and *N*-acetylhexoses. Accurate mass determination also suggest compositions with two or no fucose residues or more sialic acids than antennae. A complete overview of glycoforms confirmed in the assay performance experiments is listed in the Supporting Information Table S1. Additionally, a limited characterization has been performed on the EPO standard and the CCS EPO and is presented in a separate section at the end of the Supporting Information.

Several low abundant EPO glycosylation variants observed in the CCS material were not detectable or quantifiable in the bio-processed EPO standard. In detail, the phosphorylated high mannose glycans, the di-fucosylated structures and compositions with higher sialic acid numbers then antennae (except H6N5F1S4) were not verified in the EPO standard (see Supporting Information Table S1). Overall, the data suggests that more complex EPO glycan structures with higher mannose/sialic acid content are removed by the currently applied bio-pharmaceutical purification process.

Since minor changes in the analyte profile are expected during method optimization, especially with respect to changing sensitivity, it is not meaningful to use the complete analyte list for optimization experiments. Therefore, these were analysed using the 9 most abundant compositions and the most abundant phosphorylated high mannose glycan (Man6P; see Supporting Information Table S2). As *O*-acetylation of sialic acids was hard to quantify reliably, these peaks/glycoforms as well as salt adducts (for the high mannose glycans) are summed into a single value and reported as compositions.

With regard to the LacNAc units, literature reports the presence of mainly tri- and tetra-antennary glycans with lower abundance of bi-antennary glycans. LacNAc repeats are also well established, but limited to tri- and tetra-antennary glycans^4, 28^. Linkage isomerism with respect to the antennae is limited to tri-antennary glycans, having two antennae either on the α1,3- or α1,6-mannose, as all galactoses are linked in a β1–4 fashion^28^. Localization of one or multiple LacNAc repeats to different antennae potentially produces another form of isomerism. Complete fucosylation is reported and has been identified as α1,6-linked core fucose^17, 28^. It is well known that CHO cells usually produce exclusively α2,3-linked sialic acids. Sialic acids on EPO are known to carry *O*-acetylations and NGNA content and incomplete antenna sialylation are likewise established^4, 17, 28^. More sialic acids than antennae are also documented for EPO^29^. Phosphorylated high mannose type glycans are known to be present in minor quantities^4^. Afucosylated EPO glycans have been reported to be synthesized in CHO cells under specific conditions, so their detection at low abundance is not surprising^30^. To our best knowledge, the existence of glycans with equal amounts of hexoses and *N*-acetylhexosamines and of difucosylated glycans has not been reported so far.

### Affinity purification

The initial step of the sample preparation is key in ensuring that detected glycans derive predominantly from EPO and not from other glycoproteins present in the CCS. To this end, we used anti-EPO antibodies coupled to Sepharose beads for trapping and washing on a filter plate. Selectivity and recovery are crucial parameters.

#### Selectivity

**Figure 3:**
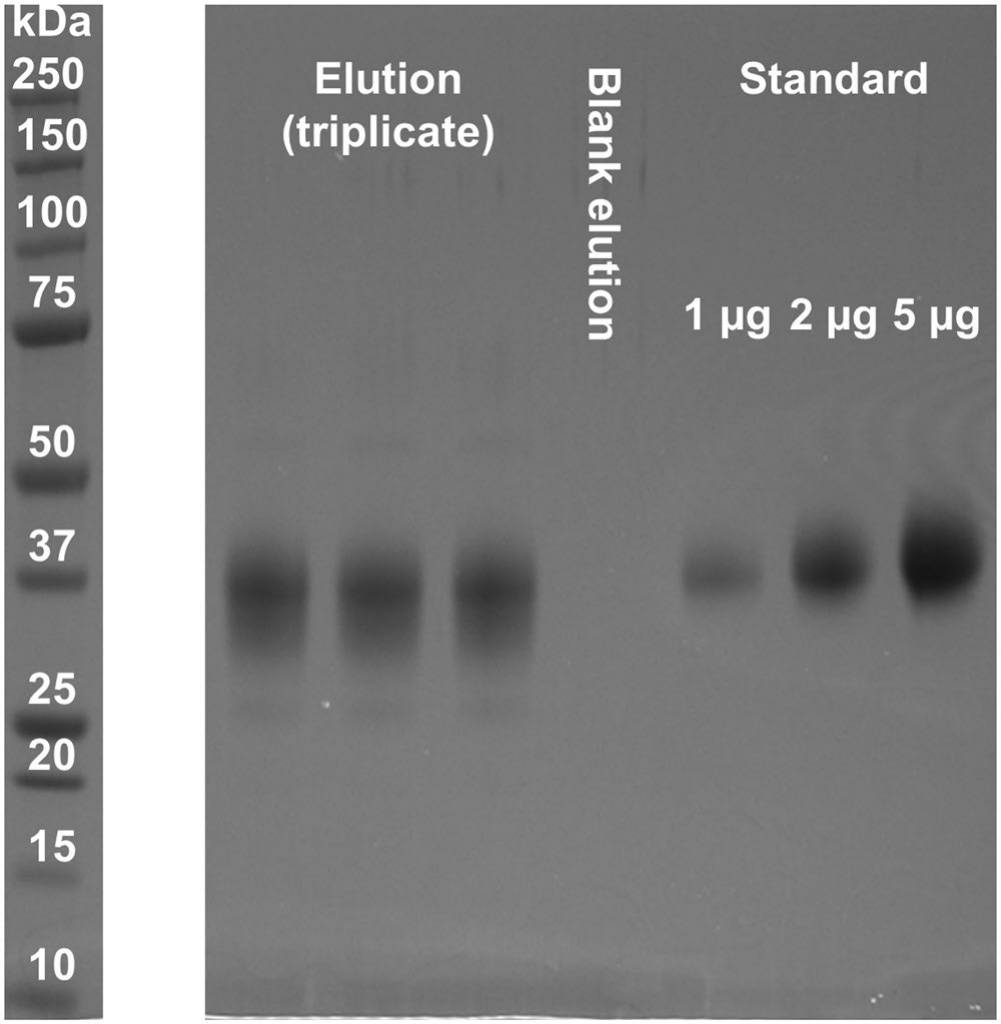
EPO purity after affinity purification. From left to right: Eluate of the affinity purification from CCS (triplicate); Eluate without prior application of CCS (negative control); EPO standard in ascending order of amounts (1, 2 and 5 µg). The three different bands of each affinity purification of CCS were excised and subjected to a standard protein identification workflow (see Supporting Information Table S3).

SDS-PAGE was employed to verify the purity of EPO after affinity purification from CCS (see Fig. 3). The EPO standard migrates close to the 37 kDa marker protein. The non-purified CCS shows the strongest intensity at a similar migration position, but expectedly contains many other proteins as well (data not shown). Likely due to the large glycosylation heterogeneity, captured EPO from CCS migrates in a very broad band. Nevertheless, the highest intensity is observed at a migration position close to the 37 kDa marker as well, albeit at a slightly lower apparent molecular weight. The observation that the band for the EPO standard is more focused may be explained by purification steps which are likely to exclude proteoforms with extreme charge and/or size. The negative control proves that no significant amount of anti-EPO antibodies are eluted under the applied purification conditions, for example due to hydrolysis of the bead-antibody linkage. A proteomics analysis of the three bands after excision and in-gel digestion confirmed that all bands contain predominantly EPO (see Supporting Information Table S3). In Supporting Information Figure S1, a MALDI-TOF-MS spectrum of a blank elution after complete work-up is shown. The highest peak detected in this negative control is below the limit of quantitation (LOQ) in the samples, proving that the affinity purification does not introduce significant contamination into the glycosylation analysis.

**Figure 4:**
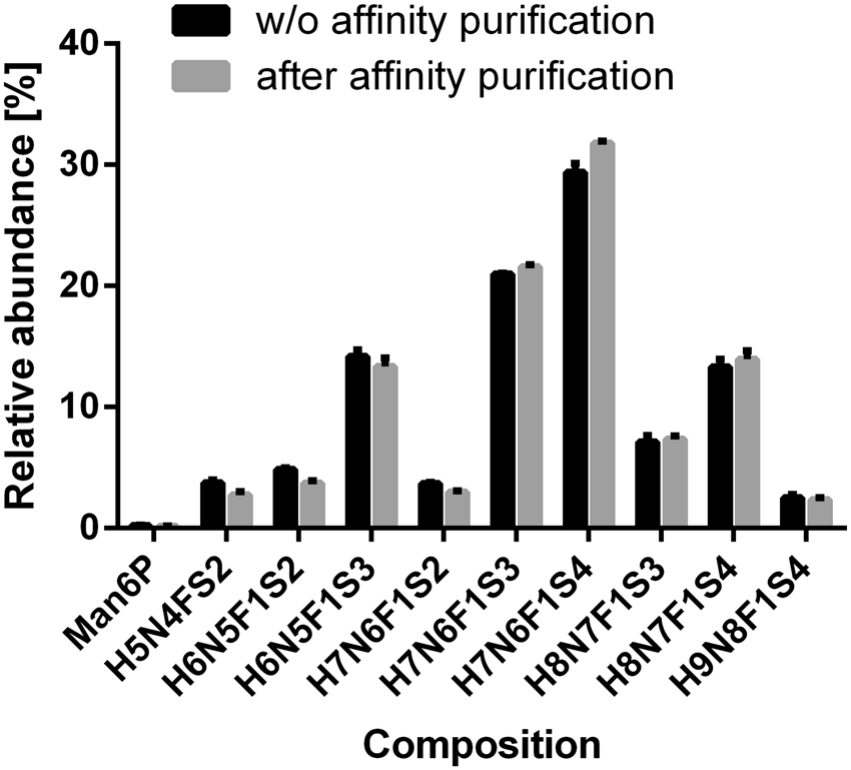
EPO composition selectivity during affinity purification. Profiles obtained with (grey) and without (black) affinity purification are highly similar. Only minor quantitative differences were observed.

Furthermore, glycosylation profiles of the EPO standard were acquired before and after affinity purification. Figure 4 demonstrates only minimal quantitative differences proving that the applied affinity purification protocol does not significantly impact the EPO glycosylation profile. Notwithstanding, unusual glycoforms with reduced anti-EPO antibody affinity might escape the capturing step. These results were independently repeated using CGE–LIF as a readout (see Supporting Information Table S4). Additionally, we repeated the affinity purification with monoclonal anti-EPO antibodies and obtained the same results (see Supporting Information Table S5). Since the specific epitope recognized by various commercially available antibodies may be different and may include glycans as epitopes, we recommend that a user of our method perform this test with their anti-EPO antibody of choice.

#### Recovery

The recovery of the affinity purification step was assessed with a BCA assay. The possibility to use the EPO standard for calibration instead of BSA increased our confidence in the obtained results. Nonetheless, due to its limited specificity the reported values should be understood as an estimation rather than exact values. A major improvement in recovery was afforded by switching the elution solvent from a 100 mM FA to a 100 mM HCl solution. This improved the recovery from 33 ± 4% (mean and standard deviation) to 54 ± 3% for the EPO standard and from 27 ± 6% to 48 ± 0.3% for the EPO purified from CCS. The change in elution solvent did not influence the glycan profile (see Supporting Information Figure S2). A minor improvement (58 ± 2% for the EPO purified from CCS) is afforded by not removing the excess bead buffer prior to binding to the beads (data not shown).

### Optimization of the sample preparation and MALDI-TOF-MS analysis

#### Simplification of the denaturation procedure

**Figure 5:**
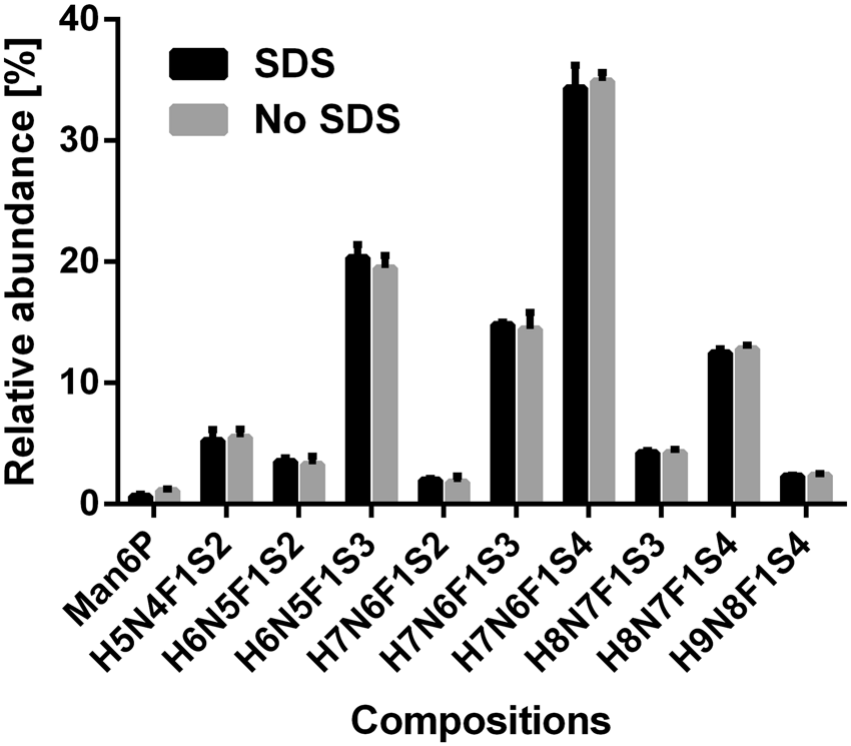
Omission of an SDS denaturation step. Omitting the additional denaturation step, which uses heating to 60 °C for 10 min in 2% SDS, did not change the EPO *N*-glycan profile. This is beneficial, as SDS may interfere with PNGaseF release and MALDI analysis. Additionally, incubation at elevated temperatures bears the risk of desialylation.

Commonly, an incubation step with detergent, often SDS, at elevated temperatures is employed to denature the target protein prior to PNGaseF release. We have previously shown that the vacuum concentration of a protein from an acidic solution, which is part of the affinity purification protocol, can be sufficient for denaturation^23^. Omitting the dedicated denaturation step reduces hands-on time, prevents addition of SDS which is known to interfere with MS performance and promotes repeatability. Importantly, a less complex sample preparation protocol is principally more robust. Moreover, sialic acids are well known to be thermolabile which is specifically crucial given their high abundance and key function in EPO. However, acknowledging that detergent denaturation is an established method in glycomics, we compared the glycosylation profiles with and without this dedicated denaturation step (see Fig. 5). No change in the qualitative and quantitative EPO glycan profile was observed and thus the technically preferable alternative of omitting the dedicated denaturation step was chosen as standard procedure. Additionally, the quality of the MALDI-TOF-MS profiles, as judged by the signal to noise ratios (S/N) for the 9 most abundant compositions and Man6P, was almost identical for both protocols (see Supporting Information Figure S3).

#### Other considerations

Increased sensitivity and the prevention of potential biases are ensured by driving the PGNaseF glycan release as close to completion as possible. Supporting Information Figure S4 demonstrates that complete release of *N*-glycans is achieved with 0.5 U of PNGaseF.

We have used 5 µg of EPO per analysis throughout the experiments reported in the manuscript, as this is already about ten times less than is usually applied for HILIC–FLD experiments^31^. This amount gives high quality spectra where many low abundant glycoforms can be detected. However, in a routine analysis, only the high abundant glycoforms may be of interest for bio-process controls and thus, it may be possible to achieve satisfactory results with lower EPO amounts. We have tested a series of decreasing total EPO quantities and found that we still obtained satisfactory results for the 9 most abundant EPO glycans at 200 ng total protein level (Supporting Information Figure S5).

### Assay performance

We also determined the precision of our method, by assessing the repeatability and intermediate precision of the whole workflow from CCS. The benefits and limitations of the information obtained on *O*-acetylation are briefly discussed. The accuracy was judged by comparison to a validated CGE–LIF method.

Our method has the advantage that it is significantly faster than conventional protocols^7^. Affinity capturing takes about 2 h and another 2 h are usually spent drying. PNGaseF digestion is performed overnight. Derivatization is achieved in under 1.5 h. SPE takes approximately 1 h for a 96-well plate, but only a few minutes for a dozen of samples. While preparing the samples for MALDI-MS analysis can take between 30 to 60 min, the measurement itself is performed within seconds per sample on our instrumentation. Automated data analysis may be as fast as 30 min on a state-of-the art server. Thus, if CCS is available at midday, results may be obtained in the early afternoon of the next day. This is sufficiently fast with respect to the weeks a fermenter is usually operated, to obtain useful information on the production in progress.

#### Repeatability and intermediate precision

**Figure 6:**
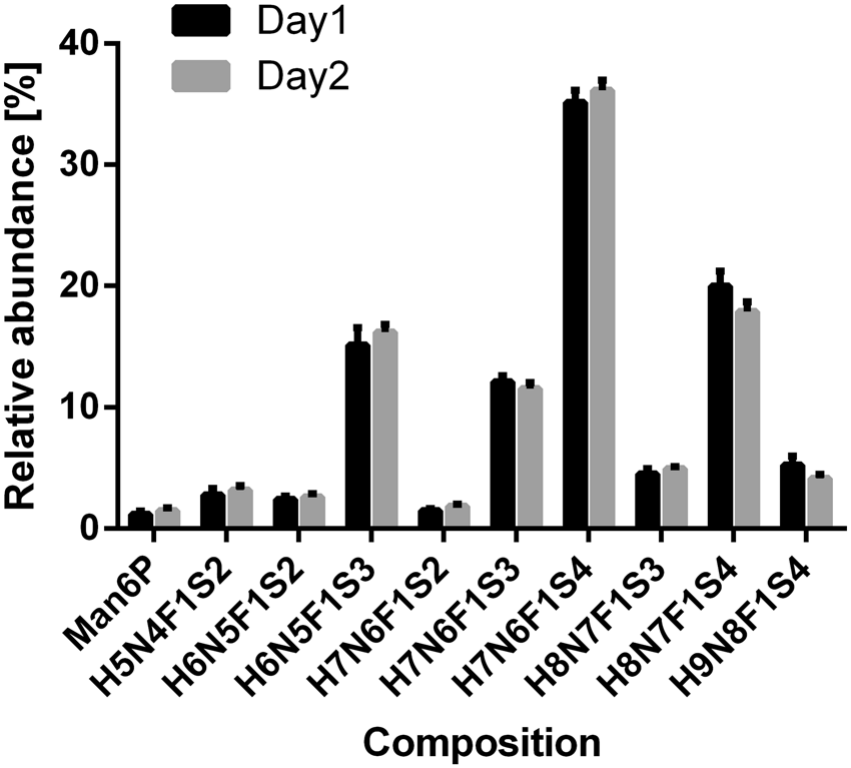
Repeatability and intermediate precision of the MALDI-TOF-MS profiling method. Relative abundances of the 9 most abundant EPO glycans and Man6P were measured in sextuplicate on two different days. Major glycans showed a CV ≤6% and there were no significant differences between the profiles of both days.

Repeatability and intermediate precision were assessed by measuring six replicates each of EPO including purification from CCS on two different days with the final method. Figure 6 shows the *N*-glycosylation of the EPO purified from CCS measured in sextuplicate on two different days and analysed using the 9 most abundant EPO glycans and Man6P. Repeatability, expressed as coefficient of variation (CV), is between 2.3% and 6.2% (with one outlier at 9.4%) for the major analytes (above 10% relative abundance). Minor glycans show CVs between 3.5% and 20.2%. No significant differences were observed between the means yielded at the different analysis days. For a comparison using all analytes (excluding *O*-acetylation) refer to Supporting information Table S1.

For the analysis of *O*-acetylation of sialic acids, the method yielded a good repeatability (RSD generally between 2% and 8%). However, the interday differences were significant, almost a factor of two in some instances. Accuracy was not assed as the CGE–LIF protocol relies on desialylation prior to glycan analysis. Details can be found in the Supporting Information in the section ‘Sialic acid *O*-acetylation analysis’.

#### Comparison of glycosylation data to a validated method

**Figure 7:**
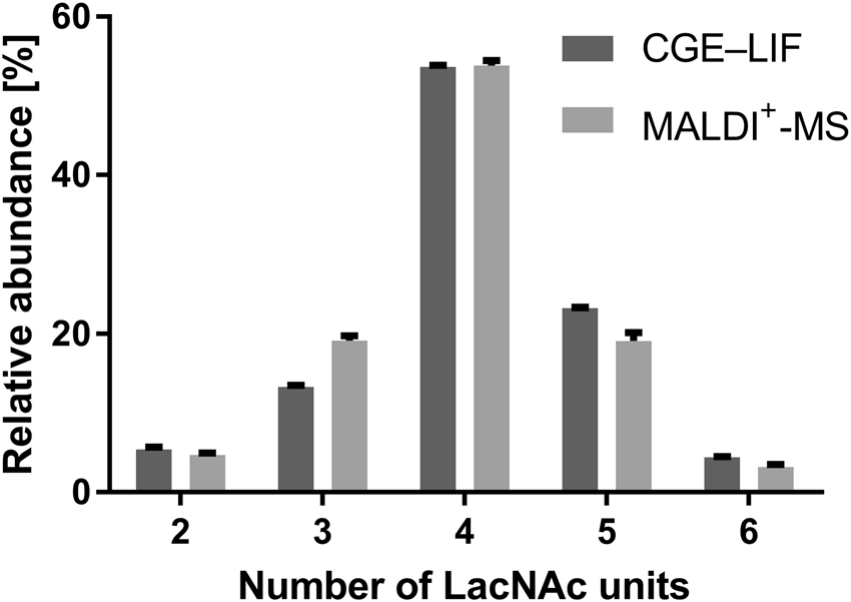
Comparison of the MALDI-TOF-MS glycan profiles to CGE–LIF profiles. Relative abundance data was summed to comparable categories based on the number of LacNAc units.

In order to judge the accuracy of the developed method, we compared the profiles of the EPO standard measured by our MALDI-TOF-MS method with data obtained by CGE–LIF analysis^26^. Because the resolution of the two approaches was very different, a summation of relative abundance was necessary to be able to compare the quantitative results. The CGE–LIF separation (after desialylation) is mainly based on antennarity. In the electropherograms, seven peaks were integrated belonging to bi-, tri- and tetra-antennary structures with no LacNAc repeats and tri- and tetra-antennary structures with one or two LacNAc repeats. While the MALDI-TOF-MS method distinguishes much more species based on the composition, it is not feasible to differentiate the isomeric structures sharing the same number of LacNAc units. Thus, all areas of glycan species with an identical number of LacNAc units were summed for each method. The results shown in Fig. 7 are overall highly comparable. However, there is a preference for species with 3 LacNAc units and a negative bias for species with 5 LacNAc or 6 LacNAc units in the MALDI-TOF-MS analysis. While the different resolution of the CGE–LIF may offer a (partial) explanation, subtle differences in response factors of the MALDI-TOF-MS detection are also consistent with the differences.

### Application to glycosylation analysis of a fermentation time course

In ten different CCS samples of a fermenter run, we found EPO glycosylation differences for most of the 9 most abundant EPO glycans. A Kruskal-Wallis test revealed differences in the relative abundances, for example, of H7N6F1S4 (p = 0.0076). We then compared each sample to the rest of the samples by a Mann-Whitney *U* test. The fermentation runs 1 and 7 exhibited higher (run 1; 42.7 ± 1.3%) [mean ± standard deviation] vs. 39.8%(1.8%); p = 0.0001) and lower (run 7 38.6%(2.0%) vs. 40.3%(1.9%); p = 0.045) H7N6F1S4 content compared to the other CCS samples. This demonstrates that our method is powerful enough to also identify minor glycosylation differences in a time course of a fermentation.

## Conclusions

We have developed a workflow which enables EPO *N*-glycosylation analysis in a fermentation time course. The workflow is faster than many existing protocols, automatable, and HT-compatible from sample preparation to quantitative data analysis. A high resolution also for low abundant EPO glycoforms was achieved. No obvious biases were incurred throughout the optimized protocol. However, it showed some limitations with respect to sialic acid *O*-acetylation.

We expect the method to be generally applicable. The general strategy (affinity purification, derivatization, purification, MALDI-MS analysis and automated data processing) has been used for glycans from many different origins, such as plasma of humans and mice and different cells and cell lines (Chinese hamster ovary, human embryonic kidney and colorectal cancer derived; partially unpublished)^21, 27, 32, 33^. Additionally, we have not observed a glycoform bias of the anti-EPO antibody, despite the large diversity of glycans observed in terms of size and charge.

Our workflow resolves most of the important glycosylation features. In fact, quantitation of sialic acids, especially in combination with a distinction of *N*-acetyl- and *N*-glycolylneuramic acids, is lacking in some state-of-the art approaches^26, 28^. In contrast, the inability of our workflow to distinguish the position of the detected LacNAc units, especially to distinguish an additional antenna from a LacNAc repeat, is certainly a limitation. How much resolution is needed and what specific isomerism is important, in the end, needs to be judged on a case to case basis. In any case, our workflow delivers robust and high resolution results compared to established glycosylation analysis methods such as CGE–LIF.

## Electronic supplementary material

Supplementary Information
