# Supplementary material for: Affinity purification of erythropoietin from cell culture supernatant combined with MALDI-TOF-MS analysis of erythropoietin N-glycosylation
Source: Sci Rep. 2017 Jul 13;7:5324. doi: 10.1038/s41598-017-05641-1 (PMC5509673; doi:10.1038/s41598-017-05641-1)
Supplement: Supplementary file 1 — Supplementary Information [file 41598_2017_5641_MOESM1_ESM.pdf]

Affinity purification of erythropoietin from cell culture supernatant combined with MALDI-TOF-MS analysis of erythropoietin *N*-glycosylation

David Falck[a,*], Markus Haberger[b], Rosina Plomp[a], Michaela Hook[b], Patrick Bulau[b], Manfred Wuhrer[a] and Dietmar Reusch[b],

[a] Center for Proteomics and Metabolomics, Leiden University Medical Center, Albinusdreef 2, 2333 ZA Leiden, The Netherlands

[b] Pharma Biotech Development Penzberg, Roche Diagnostics GmbH, 82377 Penzberg, Germany

[*]to whom correspondence should be addressed; d.falck@lumc.nl

Table S1: Glycans quantified from cell culture supernatant (CCS) erythropoietin (EPO).
Analytes are detected as $[M+Na]^+$ ions. The composition is given with the following abbreviations: H = Hexose, N = *N*-Acetylhexosamine, F = Fucose, S = *N*-Acetylneuraminic acid, G = *N*-Glycolylneuraminic acid, Ac = *O*-acetylation of a sialic acid and P = Phosphorylation of a high mannose glycan. *O*-acetylation variants were summed with the corresponding glycoforms without *O*-acetylation (The sum is referred to as compositions). Two B ions where observed as sodium adducts and are likely a result of insource fragmentation (Frag). These compositions were excluded from total area normalization and their relative areas are therefore given in parentheses. For comparison, the relative quantities observed in a representative triplicate analysis of the EPO standard are also presented. (n.d. not detected; n.q. not quantified)

**Composition**	**Exact mass $[M+Na]^+$**	**Relative abundance [%] Mean ± SD**		
		Day1	Day2	EPO standard
FragH2N2S1[a] -117 ppm	1026.338 [1026.218]	(0.50 ± 0.18)	(0.56 ± 0.08)	(0.22 ± 0.03)
H5N2P1[a] -30 ppm	1359.371 (Na salt) [1359.330]	0.21 ± 0.06	0.31 ± 0.11	n.q.
FragH3N2S2	1461.476	(0.31 ± 0.09)	(0.38 ± 0.06)	n.q.
H6N2P1[a] 11 ppm, -12 ppm	1499.442 [1499.458]; 1521.424 [1521.406] (Na salt)	1.06 ± 0.19	1.25 ± 0.18	n.q.
H5N4F1S1	2082.724	0.31 ± 0.06	0.37 ± 0.06	0.55 ± 0.03
H4N4F1S2 -14ppm (two outliers)	2193.756 [2193.725]	0.8 ± 0.2	0.77 ± 0.13	0.69 ± 0.05
H5N4F1S2	2355.809	2.5 ± 0.4	2.7 ± 0.3	∑ 3.49 ± 0.19.
H5N4F1S2Ac1	2397.820	n.d.	n.d.	part of ∑[d]
H6N5F1S1	2447.856	0.28 ± 0.05	0.31 ± 0.04	0.42 ± 0.03
H5N5F1S2	2558.888	0.54 ± 0.03	0.65 ± 0.04	0.38 ± 0.03
H5N4F1S3[b] IPQ≤0.14 AV0.11	2628.894	0.26 ± 0.01	0.35 ± 0.01	n.q.
H6N5F1S2	2720.941	2.1 ± 0.2	2.2 ± 0.2	∑ 5.14 ± 0.11
H5N6F1S2 / H6N5F1S2Ac1[b,c] IPQ≤0.14 AV0.13	2761.968 / 2762.952	n.q.	n.q.	part of ∑[d]
H7N6F1S1[b] IPQ≤0.17 AV0.15	2812.989	0.22 ± 0.01	0.28 ± 0.02	0.25 ± 0.01
H6N5S3	2847.968	0.55 ± 0.03	0.69 ± 0.04	0.36 ± 0.01
H6N6F1S2[b] IPQ≤0.14 AV0.12	2924.021	0.29 ± 0.01	0.37 ± 0.05	n.q.
H6N5F1S3	2994.026	∑ 13.3 ± 1.2	∑ 13.7 ± 0.5	12.3 ± 0.4
H6N5F1S2G1	3010.021	∑ 0.64 ± 0.10	∑ 1.06 ± 0.03	0.27 ± 0.02
H6N5F1S3Ac1	3036.037	part of ∑[d]	part of ∑[d]	n.d.

H6N5F1S2G1Ac1[b]<br>IPQ≤0.16 AV0.14	3052.032	part of ∑[d]	part of ∑[d]	n.q.
H6N5F1S3Ac2[b]<br>IPQ≤0.13 AV0.11	3078.047	part of ∑[d]	part of ∑[d]	part of ∑[d]
H7N6F1S2	3086.073	1.30 ± 0.13	1.56 ± 0.12	3.57 ± 0.08
H6N6F1S3	3197.105	0.40 ± 0.01	0.54 ± 0.02	0.28 ± 0.01
H6N6F1S2G1 and/or<br>H7N6S3[b]<br>IPQ≤0.14 AV0.12	3213.100	0.34 ± 0.04	0.36 ± 0.02	0.25 ± 0.01
H6N5F1S4	3267.111	0.48 ± 0.02	0.53 ± 0.02	0.30 ± 0.01
H7N6F1S3	3359.158	∑ 11.6 ± 0.4	∑ 11.3 ± 0.4	∑ 20.36 ± 0.07
H7N6F1S2G1[b]<br>IPQ≤0.14 AV0.12	3375.153	0.35 ± 0.11	0.51 ± 0.03	0.40 ± 0.04
H7N6F1S3Ac1	3401.169	part of ∑[d]	part of ∑[d]	part of ∑[d]
H7N6F1S3Ac2	3443.179	n.d.	n.d.	part of ∑[d]
(H7N6F1S3Ac3 /<br>H7N6S4)[c]	3485.190 / 3486.185	n.q.	n.q.	n.q.
H7N6F2S3[b]<br>IPQ≤0.14 AV0.13	3505.216	0.54 ± 0.08	0.72 ± 0.03	n.d.
H7N6F1S4	3632.243	∑ 30.8 ± 0.8	∑ 30.7 ± 0.6	∑ 28.0 ± 0.7
H7N6F1S3G1	3648.238	1.37 ± 0.26	1.74 ± 0.14	0.68 ± 0.03
H7N6F1S4Ac1	3674.254	part of ∑[d]	part of ∑[d]	part of ∑[d]
H7N6F1S4Ac2	3716.264	part of ∑[d]	part of ∑[d]	part of ∑[d]
H8N7F1S3	3724.29	∑ 4.8 ± 0.4	∑ 5.37 ± 0.13	∑ 6.1 ± 0.4
H8N7F1S3Ac1	3766.301	part of ∑[d]	part of ∑[d]	n.d.
H8N7F1S3Ac2	3808.312	n.d.	n.d.	part of ∑[d]
H8N7S4	3851.317	0.38 ± 0.06	0.36 ± 0.02	n.q.
H7N6F1S5[b]<br>IPQ≤0.13 AV0.11;<br>-10.5ppm	3905.328<br>[3905.287]	0.34 ± 0.03	0.33 ± 0.03	n.q.
H8N7F1S4	3997.375	∑ 17.5 ± 1.0	∑ 15.2 ± 0.6	∑ 12.8 ± 0.5
H8N7F1S3G1	4013.37	0.68 ± 0.17	0.79 ± 0.08	0.27 ± 0.03
H8N7F1S4Ac1	4039.386	part of ∑[d]	part of ∑[d]	part of ∑[d]
H8N7F1S4Ac2	4081.396	part of ∑[d]	part of ∑[d]	part of ∑[d]
H9N8F1S3	4089.423	0.77 ± 0.09	0.87 ± 0.04	0.86 ± 0.08
H8N7F1S5	4270.46	0.28 ± 0.03	0.25 ± 0.03	n.q.
H9N8F1S4	4362.507	∑ 4.6 ± 0.6	∑ 3.5 ± 0.3	∑ 2.3 ± 0.2
H9N8F1S4Ac1	4404.518	part of ∑[d]	part of ∑[d]	part of ∑[d]
H10N9F1S4[a]<br>13.4 ppm	4727.640<br>[4727.703]	0.46 ± 0.08	0.33 ± 0.03	n.q.

[a] Calibration at the outer edges of the mass range is challenging. Therefore, higher mass errors were accepted in these regions. Additionally, accurate masses are given for these peaks in brackets.

[b] In lower abundant analytes the isotopic pattern is more difficult to estimate. However, an average IPQ≤0.15 was observed for all of these glycans.↓

[c] Due to two compositions with only 1 Da mass difference being present, both were excluded from quantitation.
[d] Sialic acid *O*-acetylation peaks where not quantified individually. The values obtained were summed into the non-*O*-acetylated glycoform (composition).

Table S2: Short list of analytes used during method development.

Name (composition)	Exact masses $[M+Na]^+$	Glycoforms included
H7N6F1L4	3632.243; 3674.254; 3716.264; 3758.275	H7N6F1L4; H7N6F1L4Ac1; H7N6F1L4Ac2; H7N6F1L4Ac3
H7N6F1L3	3359.158; 3401.169; 3443.179	H7N6F1L3; H7N6F1L3Ac1; H7N6F1L3Ac2
H8N7F1L4	3997.375; 4039.386; 4081.396; 4123.407	H8N7F1L4; H8N7F1L4Ac1: H8N7F1L4Ac2; H8N7F1L4Ac3
H6N5F1L3	2994.026; 3036.037; 3078.047	H6N5F1L3; H6N5F1L3Ac1; H6N5F1L3Ac2
H8N7F1L3	3724.29; 3766.301	H8N7F1L3; H8N7F1L3Ac1
H9N8F1L4	4362.507; 4404.518; 4446.529	H9N8F1L4; H9N8F1L4Ac1; H9N8F1L4Ac2
H6N5F1L2	2720.941	H6N5F1L2
H7N6F1L2	3086.073	H7N6F1L2
H5N4F1L2	2355.809; 2397.820; 2439.830	H5N4F1L2; H5N4F1L2Ac1; H5N4F1L2Ac2
H6N2P1	1499.442; 1521.424 (Na salt)	H6N2P1; H6N2P1(Na salt)

Table S3: Mascot search results after in-gel digestion of purified CCS EPO.
The three bands (see Figure 2) from two separate SDS-PAGE lanes were analysed by LC–MS[2] after in-gel digestion with trypsin as described before.[1] The digests were contaminated with human keratin. As no bands matching the molecular weight of keratin were observed in the SDS-PAGE, keratin was excluded as an analytical artifact. All searches contained EPO_HUMAN (Mass:21578; erythropoietin; OS=Homo sapiens; GN=EPO) as the first (non-keratin) hit. The low sequence coverage and consequently the low scores can be explained by the abundant glycosylation of EPO (samples were not deglycosylated). Two more hits with more than one unique peptide were returned by the program: 1) Ig kappa chain C region, OS=Homo sapiens, GN=IGKC, MW=12 kDa, Score 151, two unique peptides, 34% sequence coverage (37 kDa band). 2) Neurofilament heavy polypeptide, OS=Homo sapiens, GN=NEFH, MW=113 kDa, Score 42, two unique peptides, 1% sequence coverage (25 kDa band). However, both hits distinctly do not match the weight of the MW associated with the excised bands. In addition, neither of the hits could be replicated in the duplicate sample.
The results prove that EPO is present in all three bands. We found no evidence that the 25 kDa and 50 kDa band indicate a contamination with IgG light or heavy chain, respectively. Though the 25 kDa band can be explained by low site-occupancy and/or a statistical mixture of small glycans, the 50 kDa band is hard to explain with glycan contend alone.

**Band**	**EPO was hit number**	**Score of EPO**	**Unique peptides**	**Sequence coverage**
**37 kDa**				
	1	191	7	30 %
	1	247	8	30 %
**25 kDa**				
	1	136	7	39 %
	1	132	7	39 %
**50 kDa**				
	1	143	7	26 %
	1	132	6	26 %

Anti-erythropoetin (EPO) verification for affinity purification

Polyclonal anti-EPO antibody coupled to sepharose beads were examined to check for a potential bias to particular glycan types. As described under Affinity Purification in the manuscript, EPO at drug substance level was repurified and results compared to the same sample without affinity purification. For glycan analysis of EPO, *N*-glycans were cleaved off by enzymatic treatment with PGNaseF. Released glycans were further processed with neuraminidase to clip off sialic acids. The resulting glycans were labelled with fluorescent dye 8-aminopyrene-1,3,6-trisulfonic acid (APTS) and analysed by CGE–LIF. As shown in Table S4, no differences can be seen within the method variability.

Table S4: EPO *N*-glycosylation analysed by CGE–LIF with and without affinity purification.

	Glycan types					
Samples	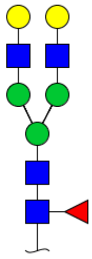	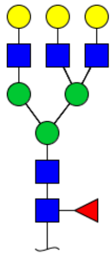	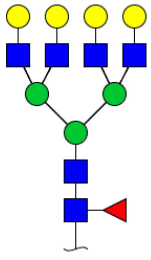	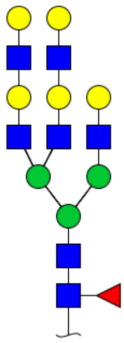	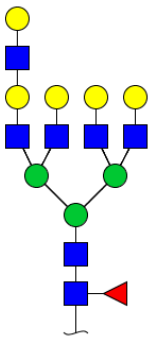	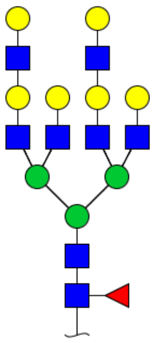
Unpurified	5.9	13.3	45.9	6.2	23.8	4.8
Affinity purified	6.7	13.4	46.3	5.8	23.2	4.6

In addition, a monoclonal anti-erythropoietin antibody was tested as described above. Monoclonal antibodies were purified from hybridoma cell culture supernatant by ammonium sulfate precipitation and ion exchange chromatography. A comparison of the results of polyclonal and the monoclonal antibody using an EPO cell culture sample are shown in Table S5.

Table S5: Comparison of CGE–LIF EPO *N*-glycosylation profiles after affinity purification with polyclonal or monoclonal antibodies.

	Glycan types					
Samples	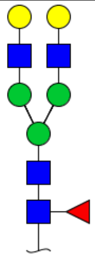	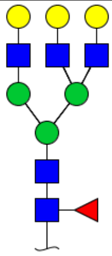	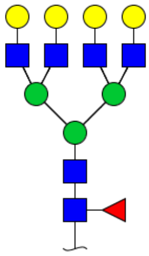	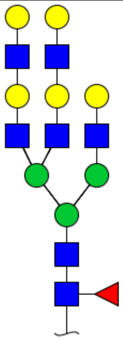	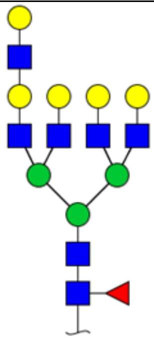	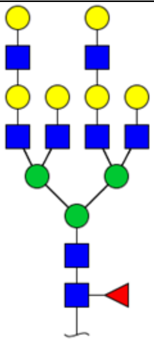
Polyclonal anti-EPO	5.8	15.1	48.8	6.1	20.3	3.9
Monoclonal anti-EPO	6.7	16.0	48.8	6.4	18.5	3.2

If commercially available anti-EPO antibodies are used a similar procedure should be followed.

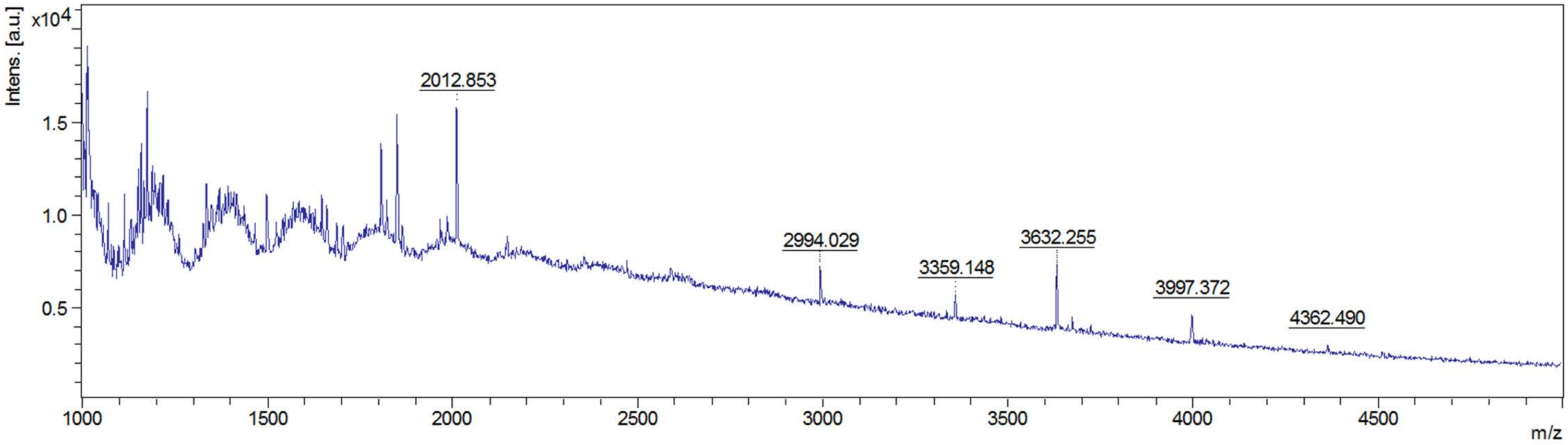

Figure S1: MALDI-TOF-MS spectrum of the negative control.

A complete work-up was performed without adding sample, after which a MALDI-TOF-MS spectrum was collected. No prominent IgG glycosylation peaks are observed which would be expected to be the main *N*-glycan contaminants incurred by the affinity purification. The minor signals observed at ca. *m/z* 3000 and above correspond to the major sample *N*-glycans and therefore likely arise from cross contamination during sample preparation, sample spotting and/or MALDI-MS analysis. The highest peak in the spectrum at *m/z* 2012.853, which may be a glycan with the composition H5N5F1, is below the limit of quantitation (LOQ). This demonstrates that the affinity purification does not interfere with the glycosylation analysis.

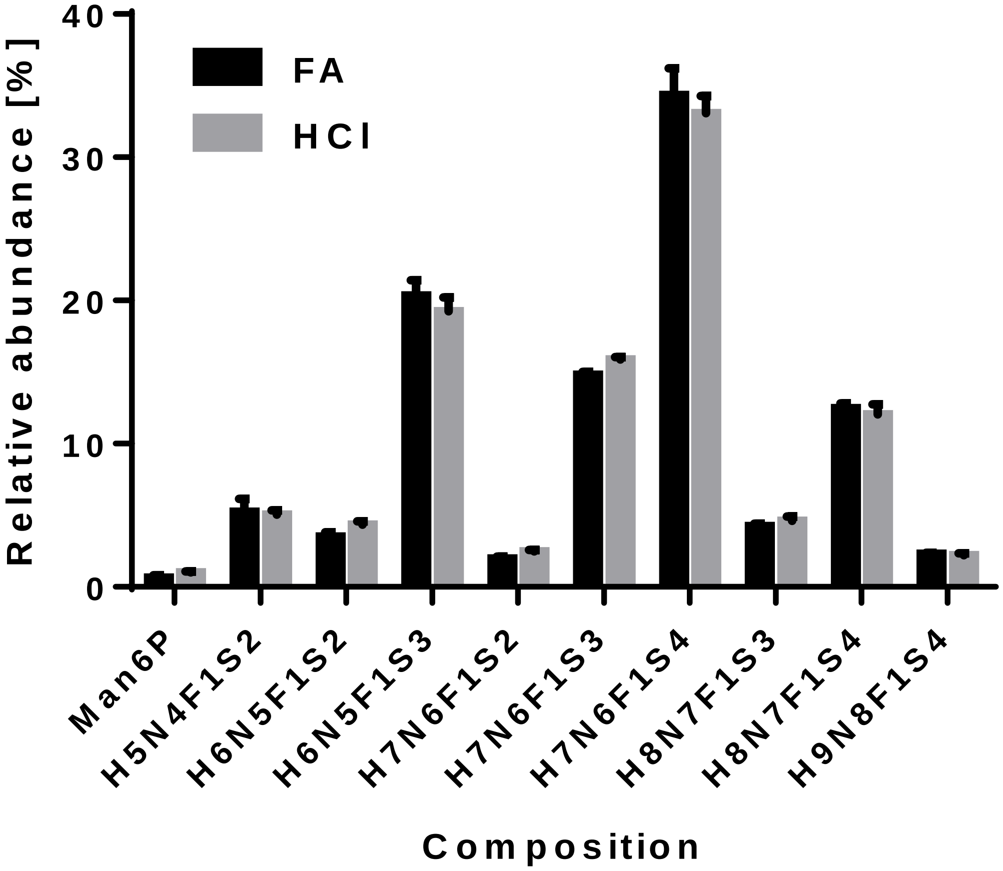

Figure S2: EPO *N*-glycan profiles comparing formic acid (FA) and hydrochloric acid (HCl) for the release of EPO from the affinity beads.

FA and HCl release are identical with respect to the shortlist profiles obtained.

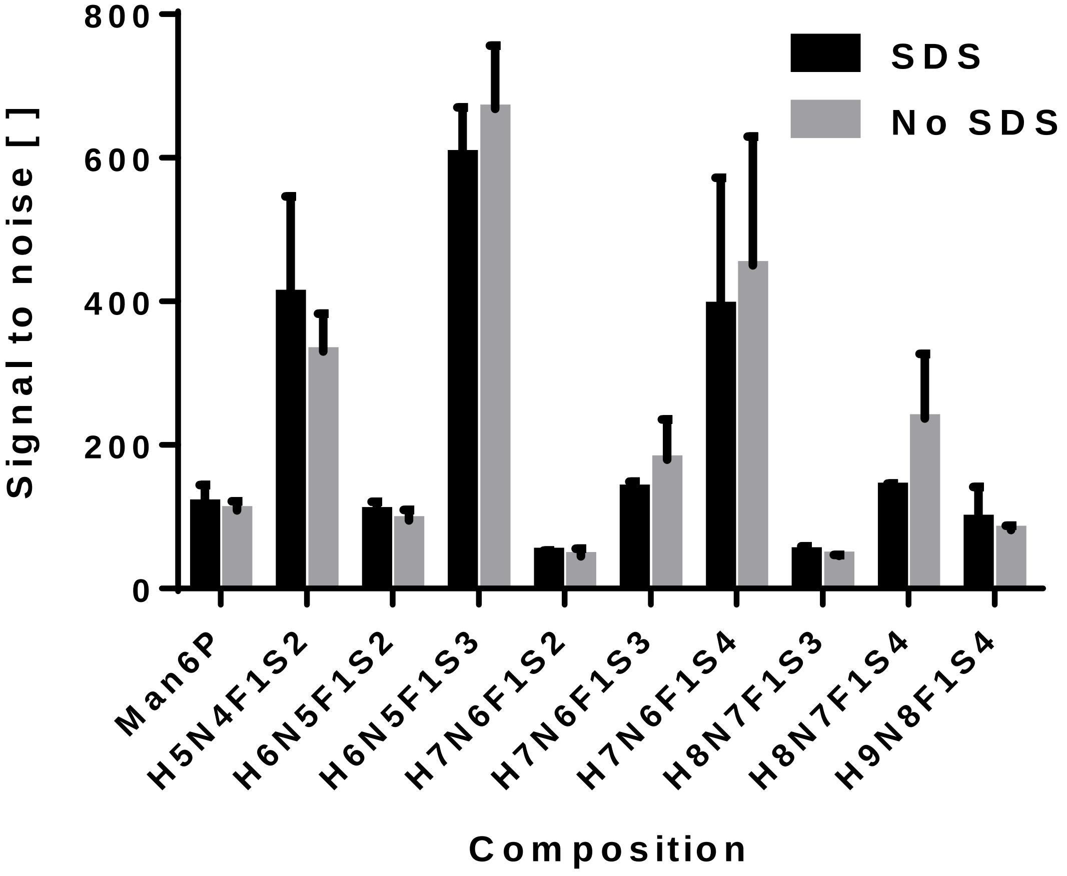

Figure S3: Signal to noise ratios (S/N) of EPO *N*-glycans comparing work-up with (SDS) and without (No SDS) a dedicated solvent-assisted denaturation step.

With both approaches identical mean S/N values are found for the shortlist glycans. S/N are a measure for the quality of the MALDI-TOF-MS data which is thus equal in both approaches.

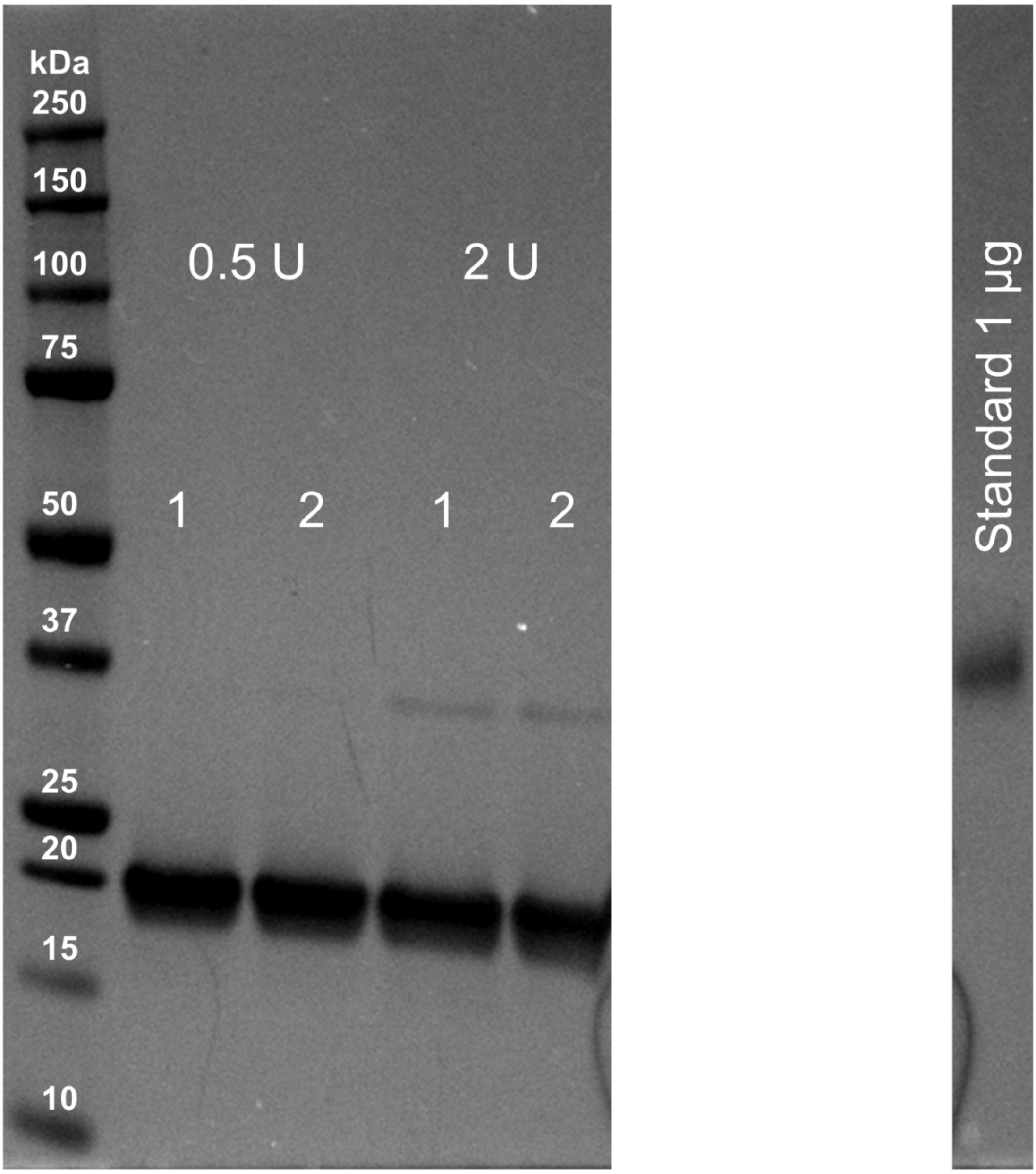

Figure S4: Completeness of the *N*-glycan release

Duplicate PNGaseF digests of the EPO standard with either 0.5 U or 2 U of PNGaseF were analysed by SDS-PAGE. Already at 0.5 U of PNGaseF for the *N*-glycan release on 5 µg of EPO, the sample shows a single relatively narrow band equivalent to approximately 20 kDa which corresponds very well with the expected *N*-deglycosylated protein. This provides strong evidence for a complete *N*-glycan release. A second band is visible in the 2 U PNGaseF samples with a molecular weight (MW) slightly lower than 37 kDa. This band is clearly distinct from the EPO standard with an apparent MW slightly higher than 37 kDa. We expect this band to be PNGaseF which fits well with the known MW of 35 kDa (UniProtKB: Q9XBM8_ELIME). Incubation with PNGaseF and SDS-PAGE were executed according the protocols described in the Materials and Methods section of the main manuscript.

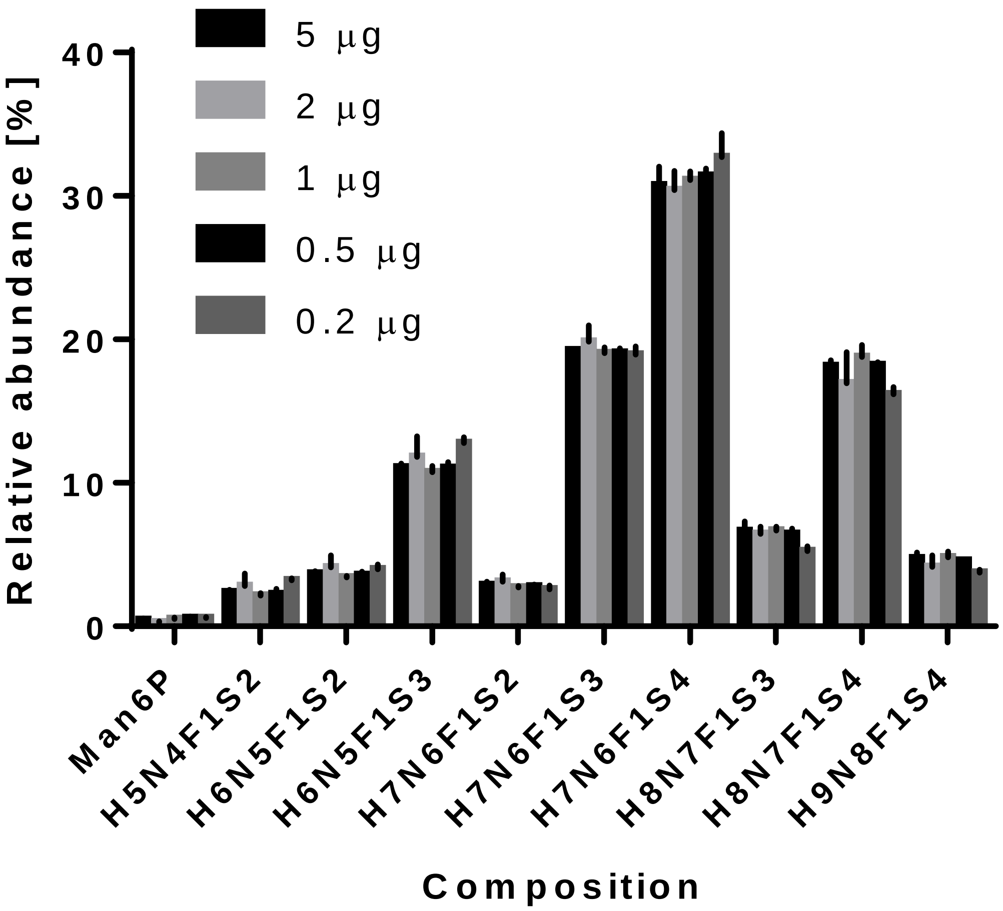

Figure S5: MALDI-TOF-MS profiles at different amounts of EPO standard.

Five different amounts of the EPO standard, namely 200 ng, 500 ng, 1.0 µg, 2.0 µg and 5.0 µg, were analysed with the final workflow without affinity purification (see Materials and Methods section, main manuscript). All tested amounts of starting material give comparable glycosylation profiles.

Sialic acid *O*-acetylation analysis

We observed between zero and two *O*-acetylations on sialylated glycans of the CCS EPO. *O*-acetylation is represented as the ratio between the *O*-acetylation variant and the non-*O*-acetylated glycoform of the same composition. Intra-day repeatability of the acetylation analysis was generally good with RSD between 2% and 8% (with the exception of three outliers, keeping in mind that most of these species are of relatively low abundance). In contrast, the inter-day comparison revealed large differences. Day 1 consistently showed a lower relative *O*-acetylation, day1 ratios being between 57% and 86% of those of day2.

Especially when normalized to the number of sialic acids, the mono-*O*-acetylated glycoforms (H6N5F1S3Ac1, H7N6F1S3Ac1, H7N6F1S4Ac1, H8N7F1S4Ac1 and H9N8F1S4Ac1) show similar ratios namely between 2.4±0.1% and 3.1±0.1% on day1 and between 3.1±0.1% and 3.7±0.1% on day2 (with one outlier 5.2±0.4% removed from day2). H8N7F1S3Ac1 presents an exception, because it has an about three times higher ratio than the other mono-*O*-acetylated glycoforms. This hints to an underlying interference or alternative composition. Interestingly, the ratios for the three di-*O*-acetylated glycoforms (H6N5F1L3Ac2, H7N6F1S4Ac2 and H8N7F1S4Ac2) are almost identical without normalization to the number of sialic acids. This can be reconciled neither with the addition of one di-*O*-acetylated sialic acid nor with the addition of two mono-*O*-acetylated sialic acids nor with a combination of both events. The ratios are between 3.2±0.2% and 3.5±0.7% for day1 and between 5.2±0.2% and 5.4±0.3% for day2. Note that the ratios for the mono-*O*-acetylated glycoforms are higher than those for the di-*O*-acetylated glycoforms without renormalization to the number of sialic acids. One *O*-acetylated species with a NGNA was quantified (H6N5F1S2G1Ac1). The ratio is unrealistic being 34±8% on day1 and 53±4% on day2. An interference or alternative composition is the most likely explanation, as this combination of low abundant modifications has not been observed for other high abundant analytes.

References

1 Plomp, R. *et al.* Site-specific N-glycosylation analysis of human immunoglobulin e. *J. Proteome Res.* **13**, 536-546 (2014).

Limited Characterization

CGE–LIF analysis of EPO standard

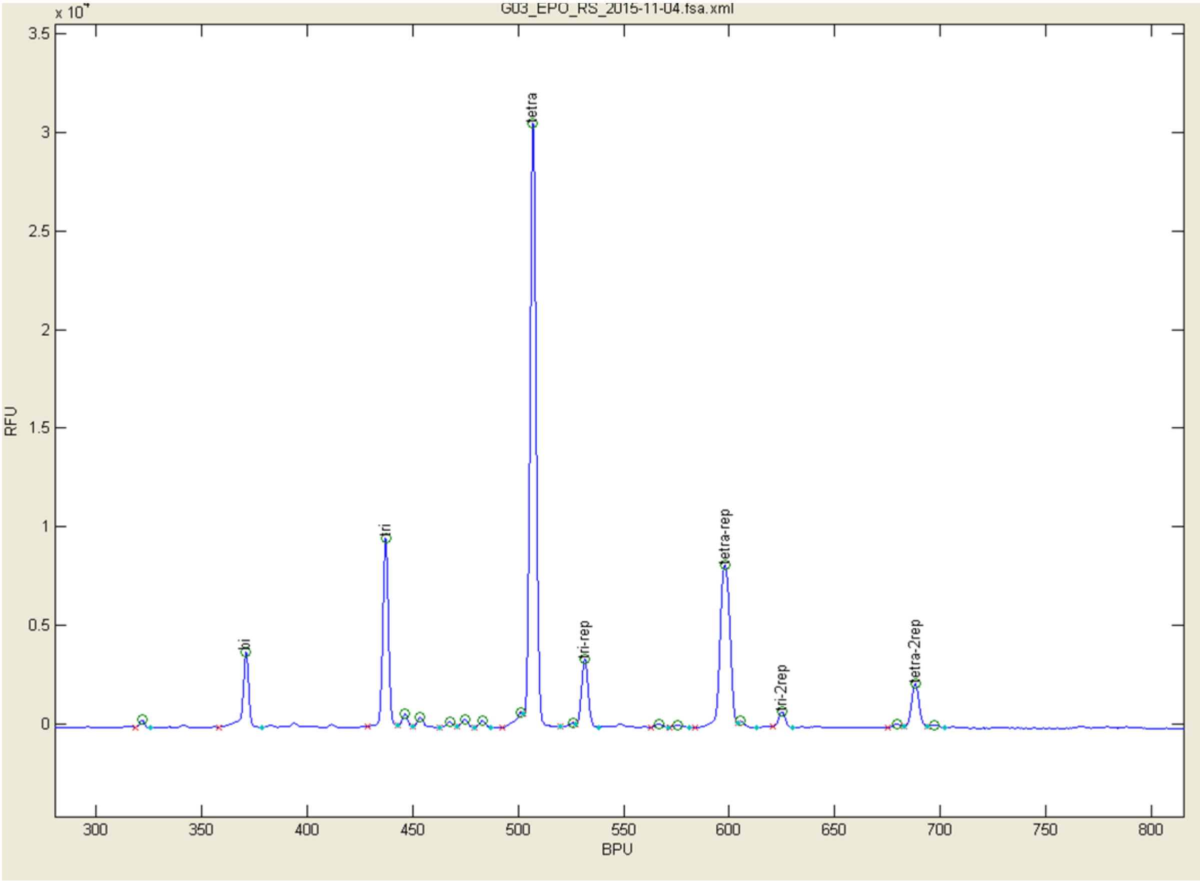

	bi	tri	tetra	tri-rep	tetra-rep	tri-2rep	tetra-2rep
EPO standard (1)	5.3	13.0	47.9	5.8	21.9	1.5	4.6
EPO standard (2)	5.7	13.4	47.8	5.8	21.5	1.5	4.4
EPO standard (3)	5.2	13.5	47.8	5.9	21.6	1.5	4.4
EPO standard (4)	5.0	13.4	48.0	6.0	21.8	1.5	4.4
EPO standard (5)	5.6	13.4	47.6	6.0	21.5	1.5	4.4
EPO standard (6)	5.8	13.2	47.6	5.9	21.6	1.5	4.4

Notes on MALDI-MS/MS

- MS/MS spectra were collected in the LIFT mode of the UltraFlextreme (Bruker Daltonics).
- MS/MS spectra are recalibrated using the exact masses of the oxonium ions *m/z* 226.0686, 388.1214, 550.1742 and 661.2063 and of the precursor ion
- Since mass accuracy was low in MS/MS mode, fragments were assigned using the unit mass.

Highlights

- Reducing end fragmentation is consistent with core fucosylation; no evidence for antenna fucosylation is observed.
- Antenna fragmentation (oxonium ions) suggests at maximum one LacNAc repeat per antenna
- For all other cases of isomerism, a random isomer is selected partially based on likelihood

_m/z_ 2355.809

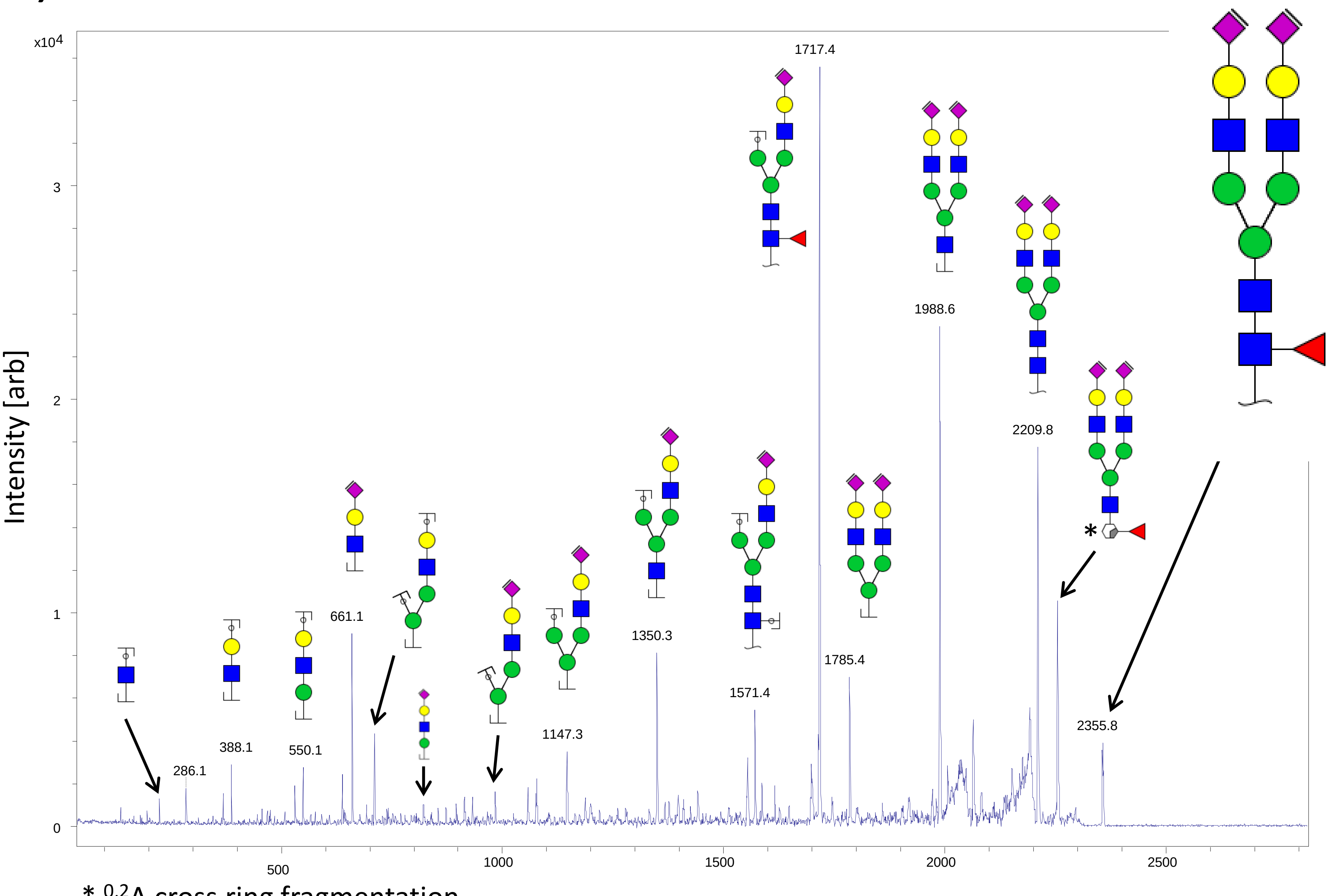

* ^{0,2}A cross ring fragmentation
of the reducing end *N*-acetylglucosamine

_m/z_ 2720.941

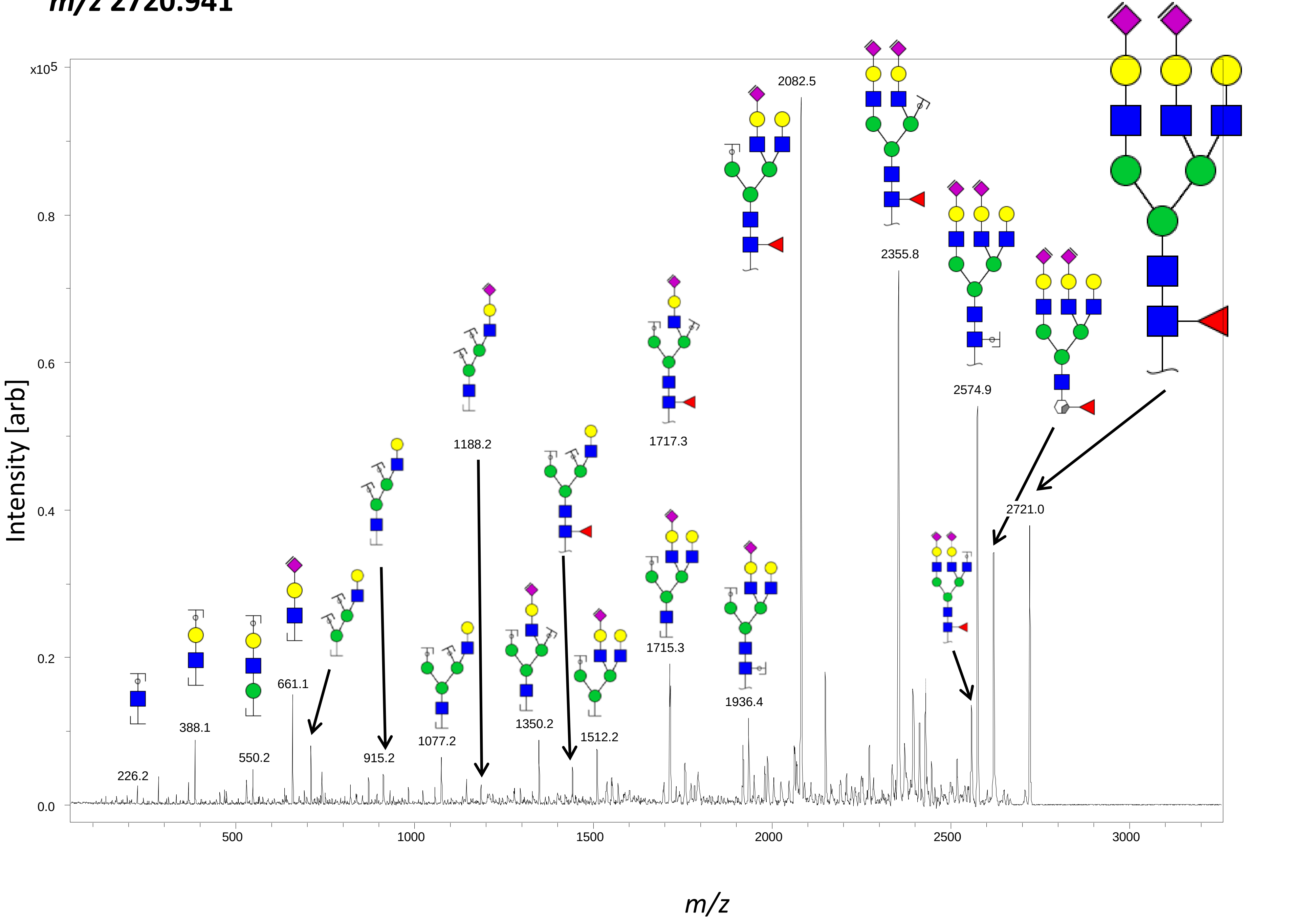
x10^5
0.0
0.2
0.4
0.6
0.8
Intensity [arb]
500
1000
1500
2000
2500
3000
m/z
226.2
388.1
550.2
661.1
915.2
1077.2
1188.2
1350.2
1512.2
1715.3
1717.3
1936.4
2082.5
2355.8
2574.9
2721.0

***m/z* 2994.026**

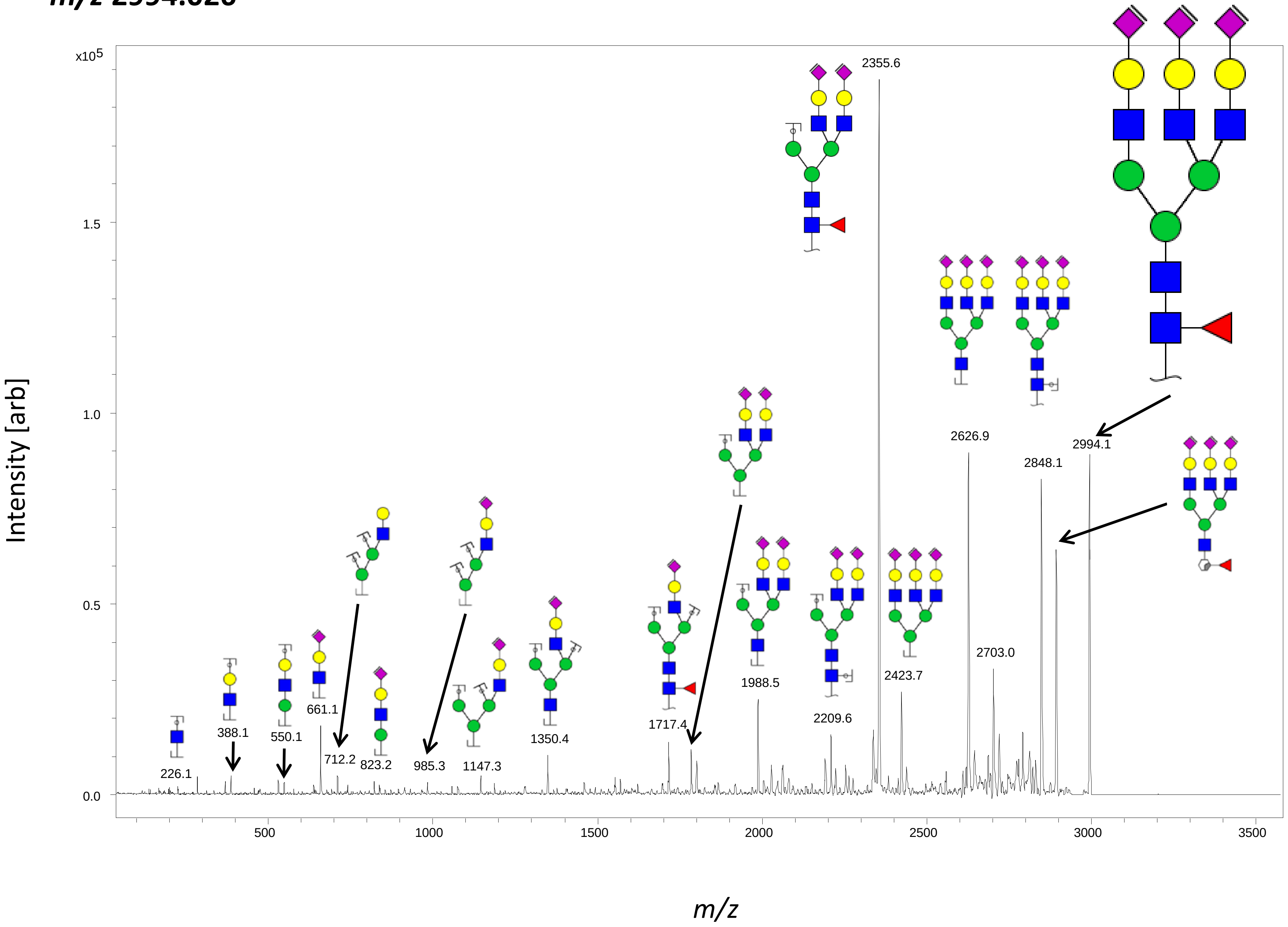
x10^5
1.5
1.0
0.5
0.0
Intensity [arb]
500
1000
1500
2000
2500
3000
3500
m/z
226.1
388.1
550.1
661.1
712.2
823.2
985.3
1147.3
1350.4
1717.4
1988.5
2209.6
2355.6
2423.7
2626.9
2703.0
2848.1
2994.1

m/z 3036.037

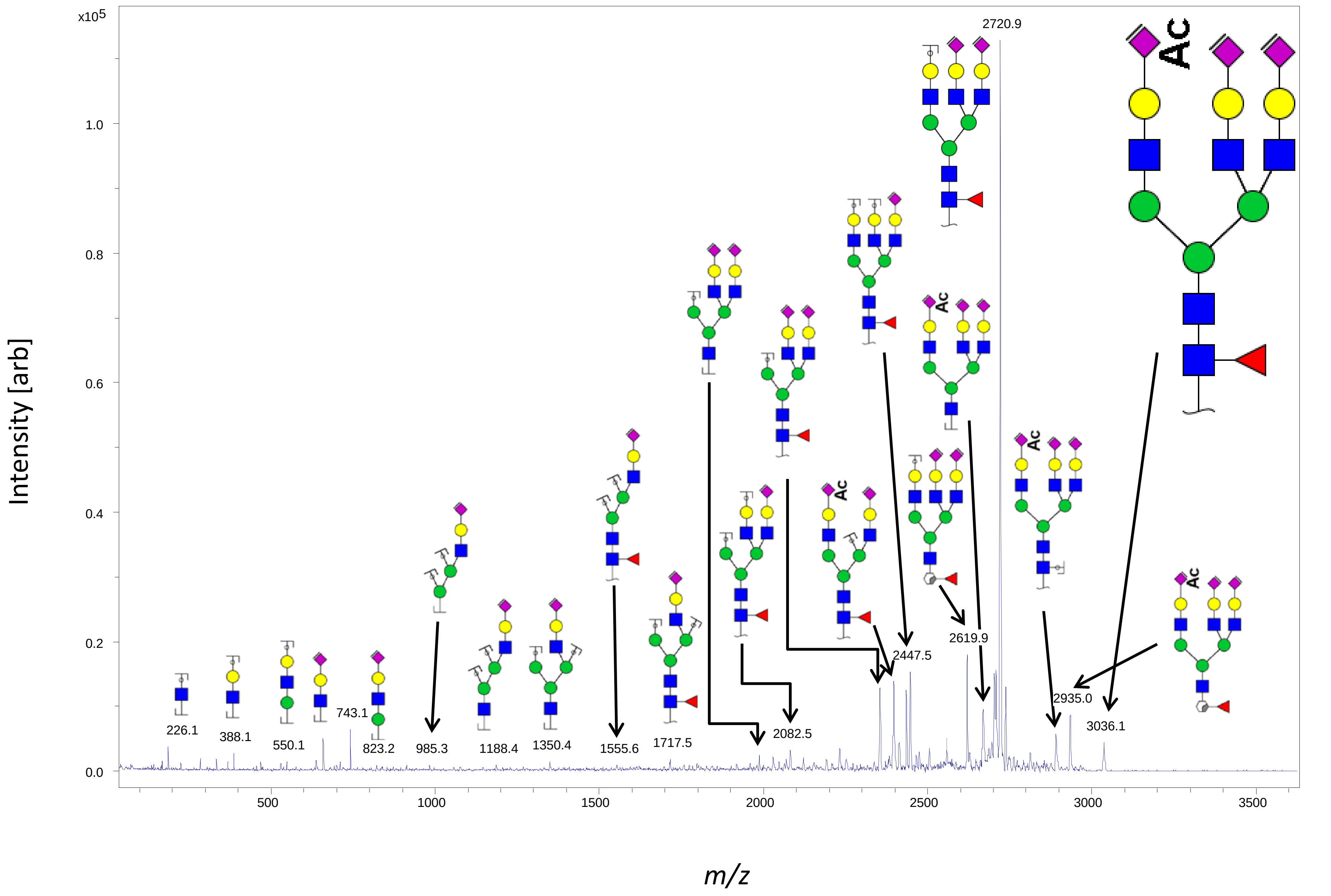

x10^5
1.0
0.8
0.6
0.4
0.2
0.0
Intensity [arb]
500
1000
1500
2000
2500
3000
3500
m/z
226.1
388.1
550.1
743.1
823.2
985.3
1188.4
1350.4
1555.6
1717.5
2082.5
2447.5
2619.9
2720.9
2935.0
3036.1
Ac

m/z 3086.073

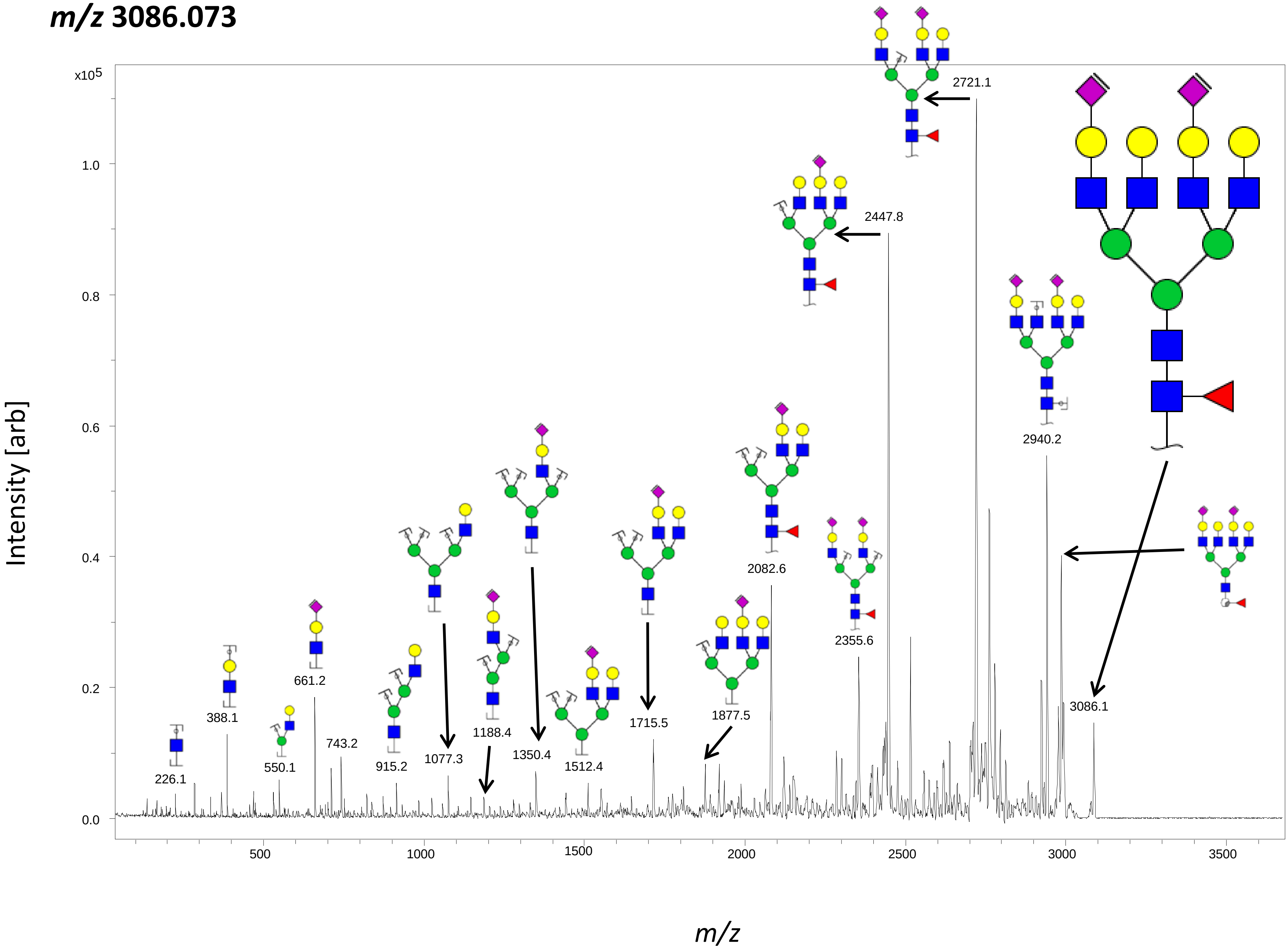
x10^5
1.0
0.8
0.6
0.4
0.2
0.0
Intensity [arb]
500
1000
1500
2000
2500
3000
3500
m/z
226.1
388.1
550.1
661.2
743.2
915.2
1077.3
1188.4
1350.4
1512.4
1715.5
1877.5
2082.6
2355.6
2447.8
2721.1
2940.2
3086.1

m/z 3359.158

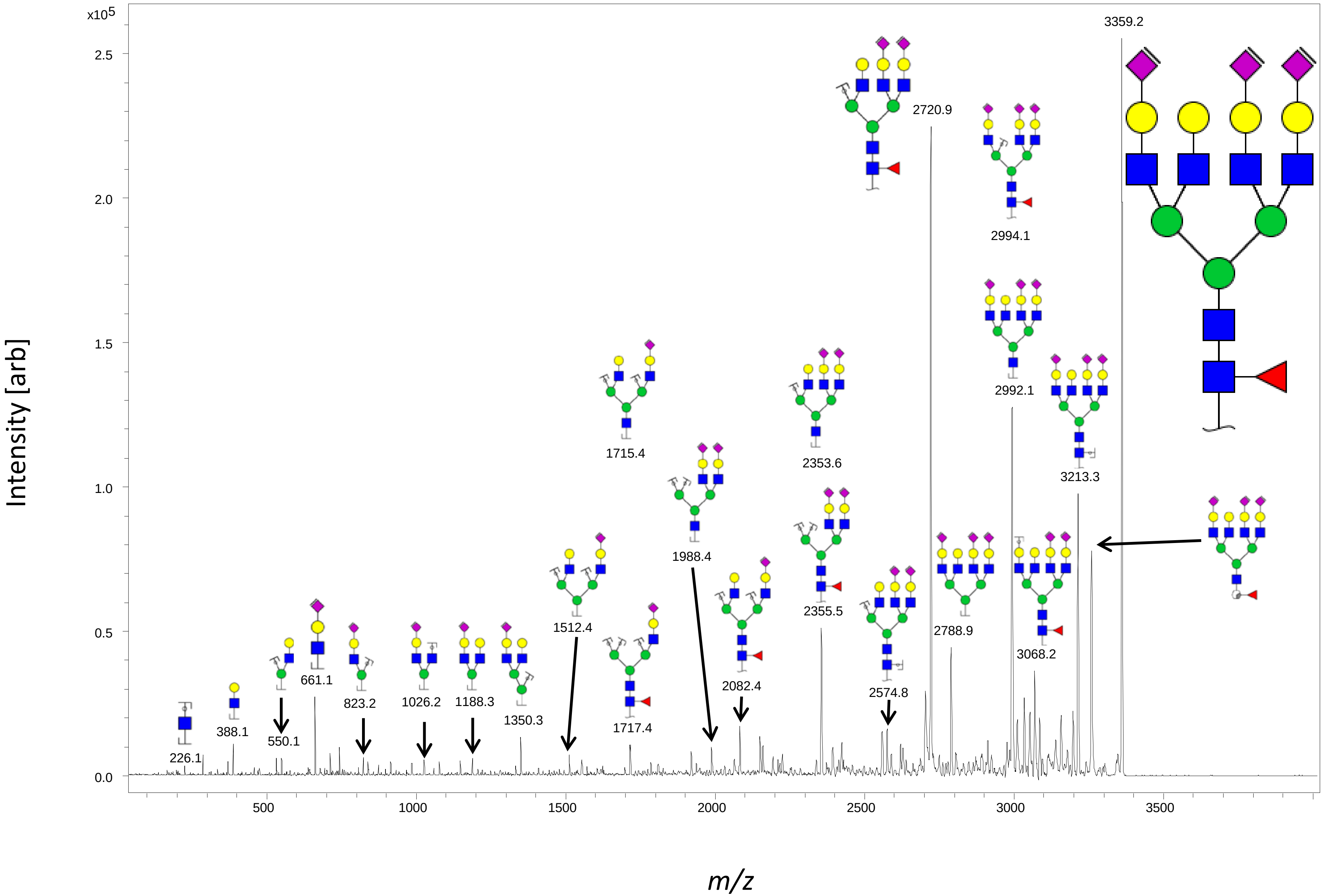

x10^5
2.5
2.0
1.5
1.0
0.5
0.0
Intensity [arb]
500
1000
1500
2000
2500
3000
3500
m/z
226.1
388.1
550.1
661.1
823.2
1026.2
1188.3
1350.3
1512.4
1715.4
1717.4
1988.4
2082.4
2353.6
2355.5
2574.8
2720.9
2788.9
2992.1
2994.1
3068.2
3213.3
3359.2

m/z 3401.169

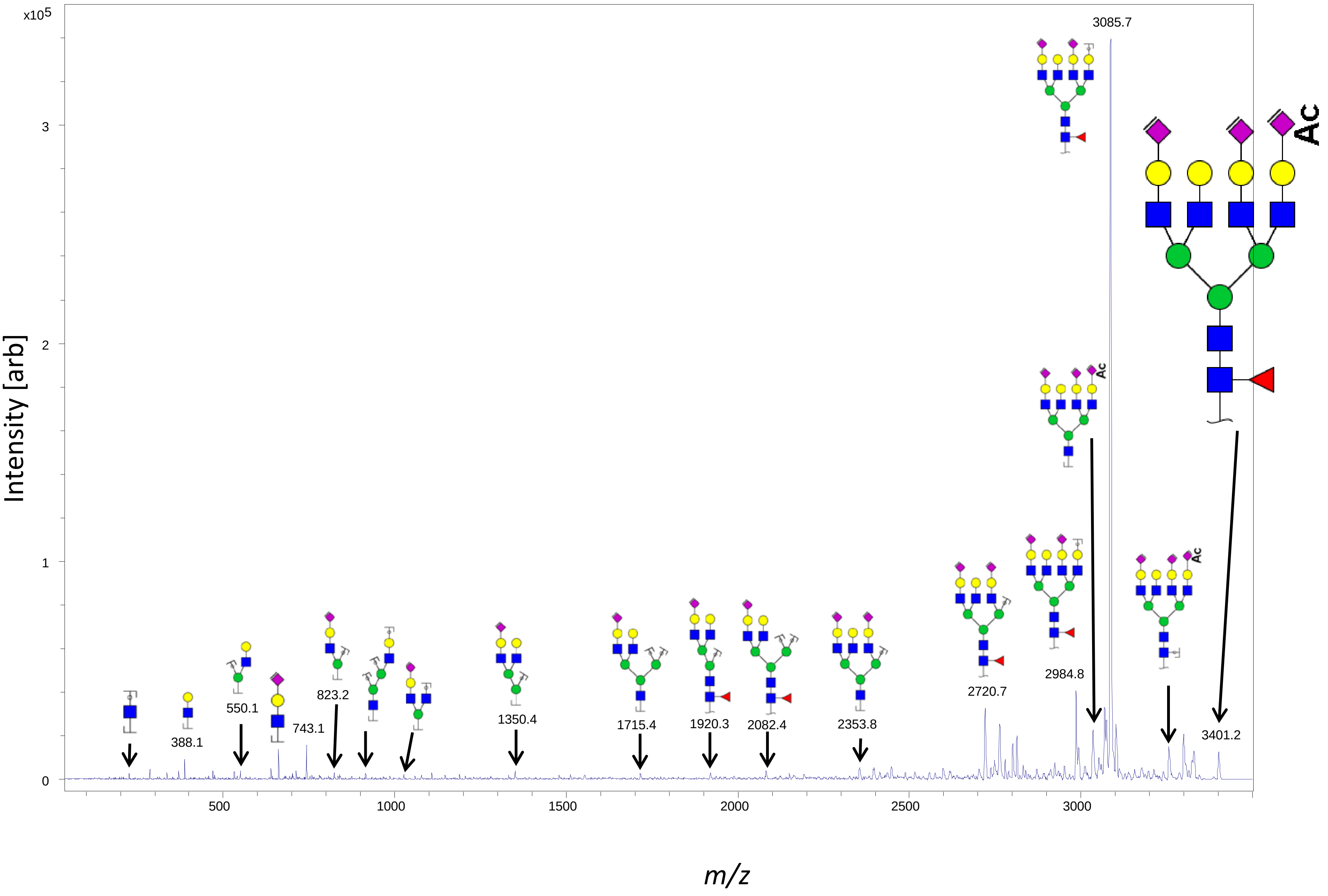
x10^5
3
2
1
0
Intensity [arb]
3085.7
Ac
388.1
550.1
743.1
823.2
1350.4
1715.4
1920.3
2082.4
2353.8
2720.7
2984.8
3401.2
500
1000
1500
2000
2500
3000
m/z

m/z 3632.243

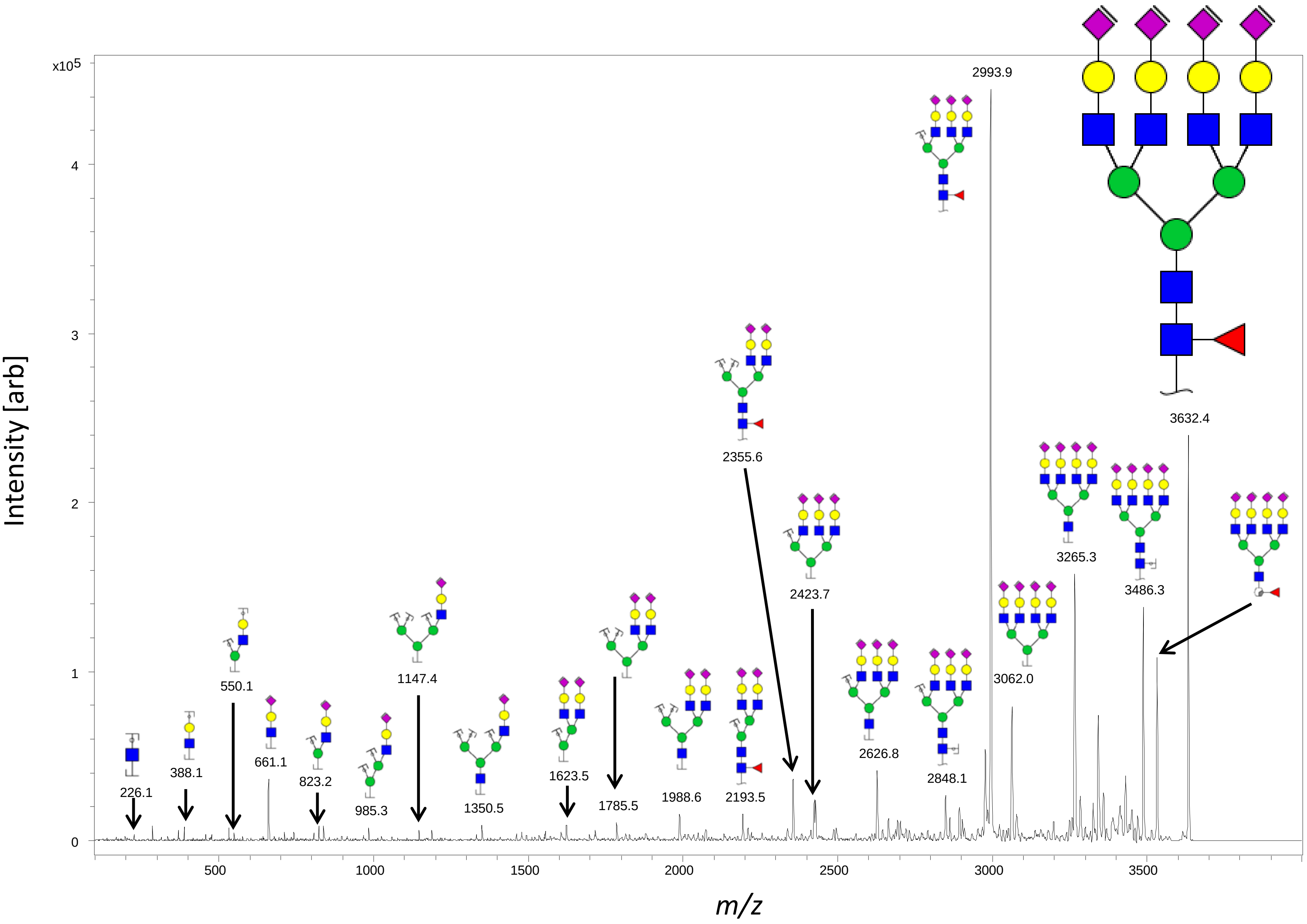
x10^5
4
3
2
1
0
Intensity [arb]
2993.9
3632.4
2355.6
2423.7
3265.3
3486.3
3062.0
2626.8
2848.1
550.1
1147.4
661.1
388.1
226.1
823.2
985.3
1350.5
1623.5
1785.5
1988.6
2193.5
500
1000
1500
2000
2500
3000
3500
m/z

***m/z* 3674.254**

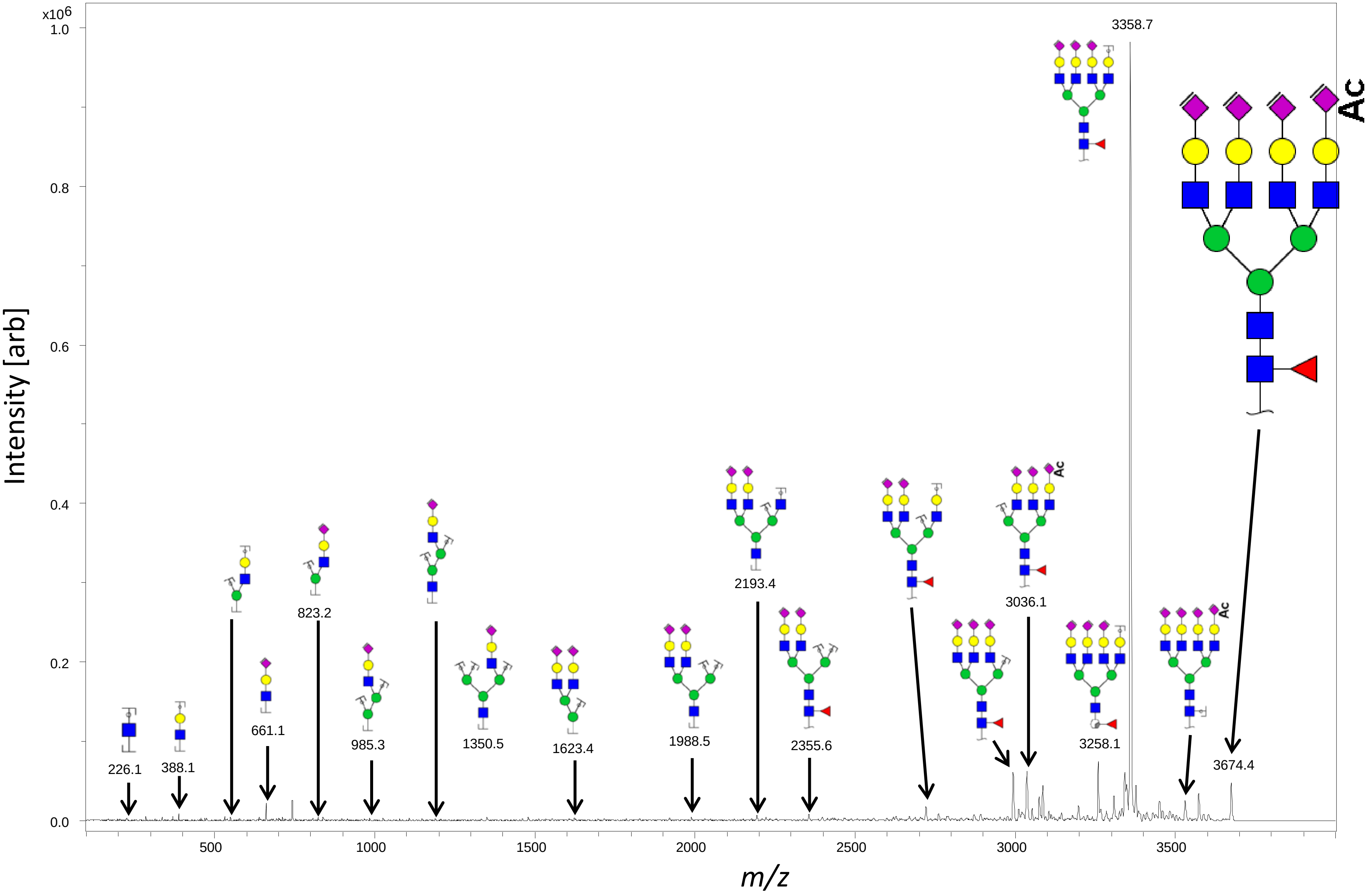
x10^6
1.0
0.8
0.6
0.4
0.2
0.0
Intensity [arb]
226.1
388.1
661.1
823.2
985.3
1350.5
1623.4
1988.5
2193.4
2355.6
3036.1
3258.1
3358.7
3674.4
Ac
500
1000
1500
2000
2500
3000
3500
m/z

m/z 3997.375

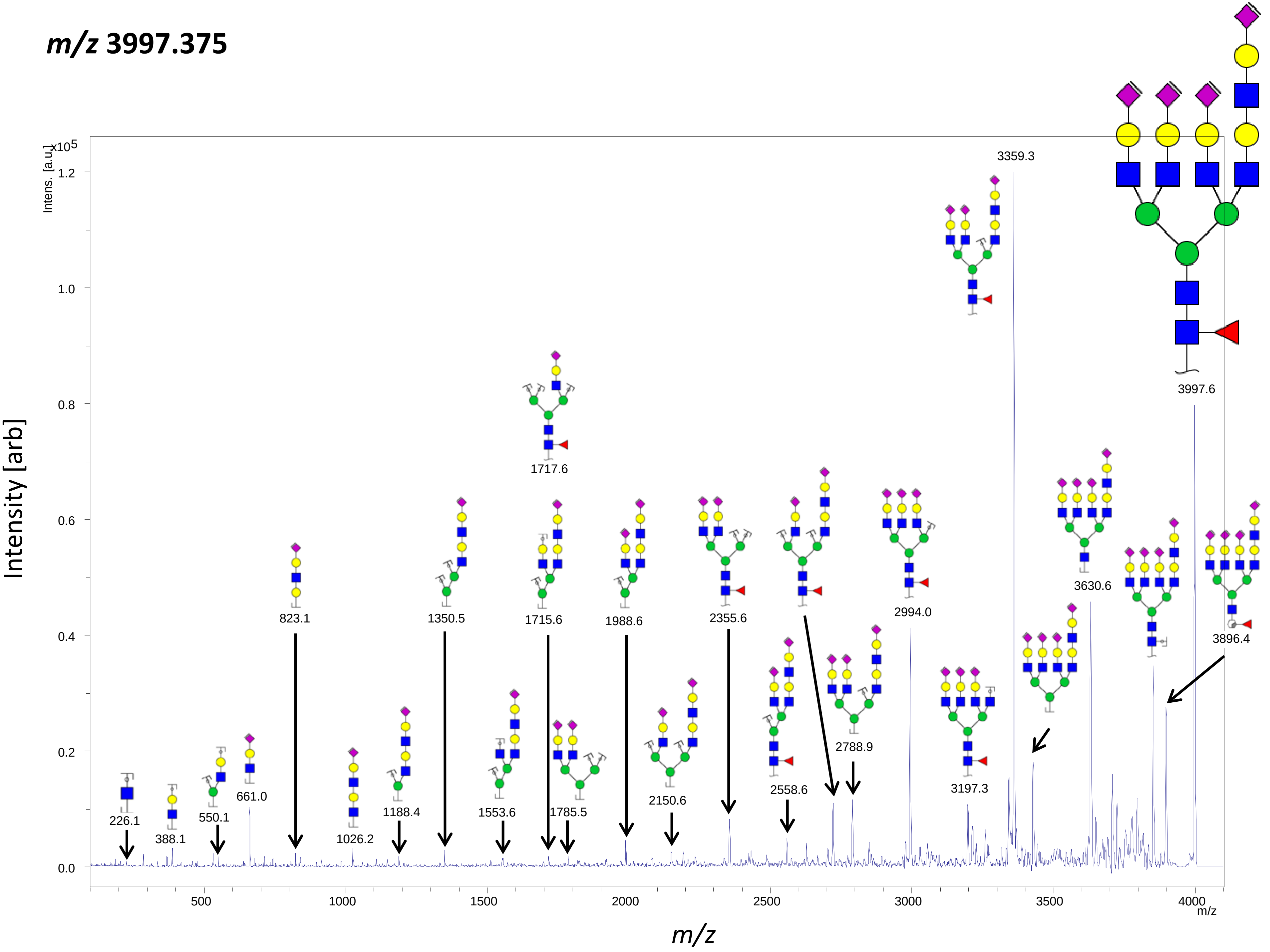

x10^5
Intens. [a.u.]
Intensity [arb]
0.0
0.2
0.4
0.6
0.8
1.0
1.2
226.1
388.1
550.1
661.0
823.1
1026.2
1188.4
1350.5
1553.6
1715.6
1717.6
1785.5
1988.6
2150.6
2355.6
2558.6
2788.9
2994.0
3197.3
3359.3
3630.6
3896.4
3997.6
500
1000
1500
2000
2500
3000
3500
4000
m/z
m/z

m/z 4362.507

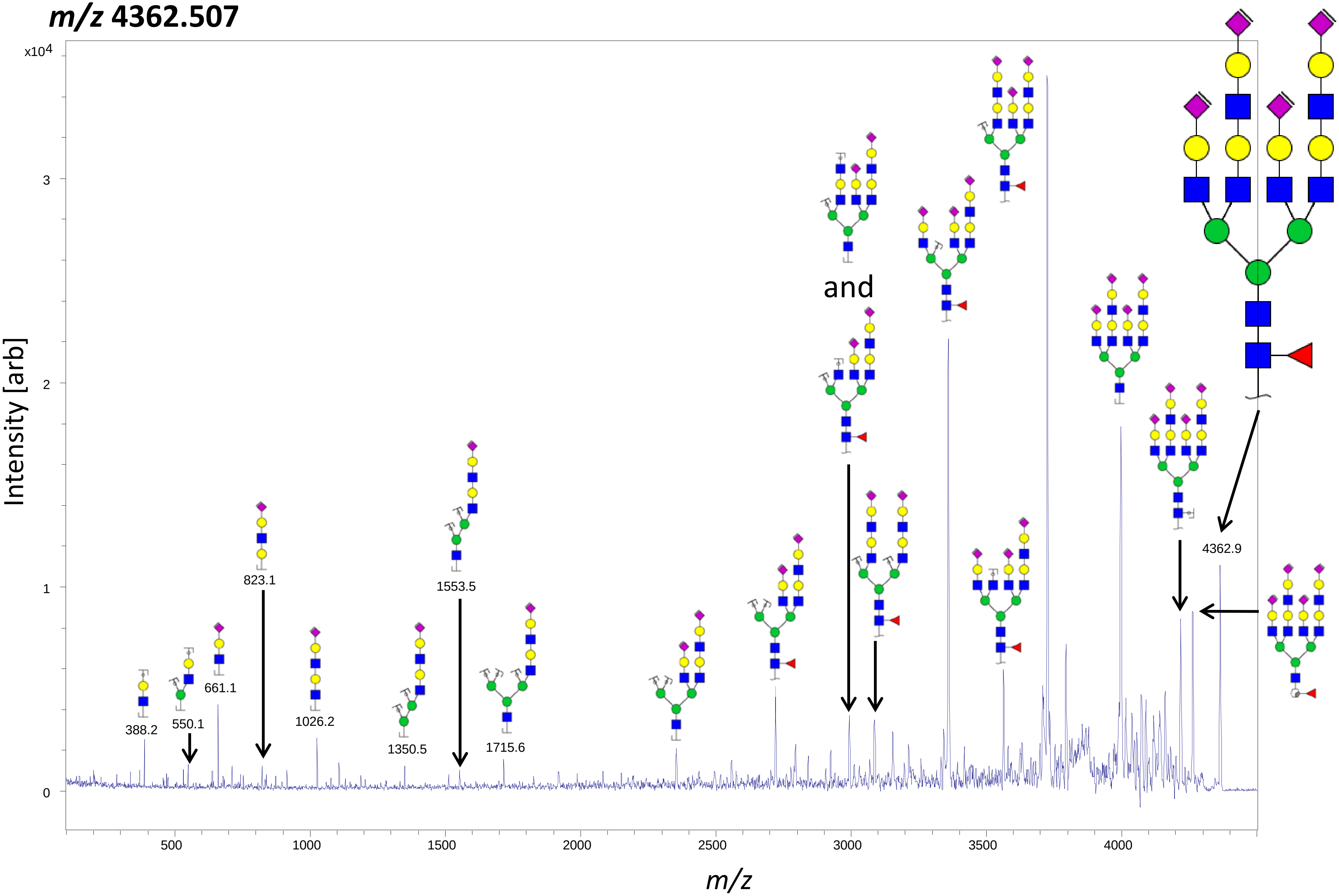

Above m/z 2000 all assignments are based on average mass, as the monoisotopic mass is not resolved
